# 4D sensor perception in relativistic image processing

**DOI:** 10.1038/s41598-025-89507-x

**Published:** 2025-02-18

**Authors:** Simone Müller, Dieter Kranzlmüller

**Affiliations:** 1 Leibniz Supercomputing Centre (LRZ), Center for Virtual Reality and Visualisation (V2C), Munich, 85748 Germany; 2 Faculty of Computer Science, Ludwig-Maximilians-Universität (LMU), Munich, 80538 Germany

**Keywords:** 4D Sensor Perception, 4D Informationen, Relativistic Image Processing, Schlingel Diagram, Environmental impact, Environmental sciences, Engineering, Mathematics and computing, Physics

## Abstract

This article introduces the 4D sensor perception in relativistic image processing as a novel way of position and depth estimation. Relativistic image processing extends conventional image processing in computer vision to include the theory of relativity and combines temporal sensor and image data. In consideration of these temporal and relativistic aspects, we process diverse types of information in a novel model of 4D space through 10 different degrees of freedom consisting of 4 translations and 6 rotations. In this way, sensor and image data can be related and processed as a causal tensor field. This enables the temporal prediction of a user’s own position and environmental changes as well as the extraction of depth and sensor maps by related sensors and images. The dynamic influences and cross-sensor dependencies are incorporated into the metric calculation of spatial distances and positions, opening up new perspectives on numerous fields of application in mobility, measurement technology, robotics, and medicine.

## Introduction

Sensor perception of modern machines is a common and important precondition for safe interaction and locomotion in complex environments^1,2^. For example, a moving vehicle needs to observe its environment and be aware of objects and changes around it to make accurate predictions and decisions according to its tasks^3,4^. This requires a depth perception in which 3D structures of the environment are reproducible. In addition to depth perception, the vehicle’s positioning and motion must be precise and predictable to react to changing situations and avoid physical harm^3,5^.

Various types of sensors can be used to extract the necessary information in such complex and interacting environments. In this context, the environmental perception consists of two aspects: a precise and fast visual recognition for the 3D projection of the environment^6^ and the positional orientation within the projected virtual environment^1^. Usually, optical sensors can be used to obtain a 3D projection of the environment. For example, sensors such as cameras, LiDARs, and Time-of-Flight (ToF) provide the visual information content to extract depth distances that enable us to render entire point clouds^3^. In turn, sensor-acquired magnetic forces, accelerations, or angular velocities can be used to estimate positions, motions, planning, navigation, and localization^7–9^. The perception of one’s own position, which is dependent on the geometric relationships of the environment, requires a precise perception of depth. This involves an understanding of kinematics, dynamics, and motion-relevant mechanisms, which are essential for the correct estimation of depth distances^3,10^.

The complexity of sensor perception arises from various lighting, contrast, weather conditions, as well as dynamic and temporal aspects associated with the objects within the environment^4,11–13^. Particularly the dynamics and associated time must be emphasized here, as the perspective and positional relation between the objects change within the environment. Requirements such as maintaining safety distances, avoiding collisions due to moving objects, and calculating the shortest possible times at minimal energy consumption must be considered^4,5,9^. In addition, individual sensors can exhibit inaccuracies, noise, consumption, or integration errors, satellite signals cannot be used everywhere and several sensors must be correctly fused which can be a challenge^3,11^.

Environmental changes captured by sensor and image data are related in space and time. The data processing requires the spatio-temporal alignment to match multiple sensor information and transfer it from a sensor-intrinsic view to a higher-order and extrinsic inertial system^14,15^. The associated temporal aspects of environmental physics and computer science can affect the depth calculation but also position estimation qualitatively.

**Fig. 1:**
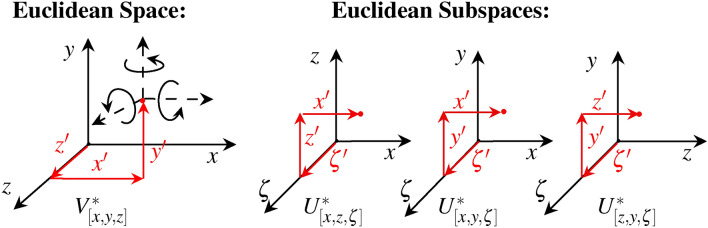
The Euclidean space can be divided into subspaces to consider temporal aspects of information. $V^{*} [x,y,z] \in \mathbb {R}^{3}$ has a undetectable temporal change. $U^{*} [x,y,\zeta ][x,z,\zeta ][z,y,\zeta ] \in \mathbb {R}^{3}$ extends the Euclidean space and enables a spatio-temporal view.

If we want to consider all temporal aspects of information, a single coordinate system within Euclidean space is not sufficient. Rather it is necessary to define temporal subspaces with a respective temporal axis $\zeta$. Fig. 1 shows our mathematical approach of temporal subspaces in Euclidean representation.

Fig. 1 shows the complexity if we consider temporal aspects in form of Euclidean subspaces. It is not trivial to understand correlations at first glance since a higher parameterization goes hand in hand with each additional dimension. This is accompanied by the curse of dimensionality, where the number of unknowns grows exponentially with the number of dimensions^16,17^. Depending on the representation, this requires several subspaces to complete the interrelated information. Further, the determination of tensors and vector invariants^18^, which describe the vector field of higher-order motions behind these models, proves to be complex, meaning that it is simply avoided in rigid-body kinematics^16,19^. In addition, these solutions require a high level of computing power for implementation^5,20^. The geometric problem of motion systems with variable poses around coordinates is hidden behind these computational operations since the general solutions are often not unique which means that there is a finite number of solutions for non-redundant ones^20,21^.

In recent years, tensor-based modeling has been introduced for a more precise description of kinematics and resulting positions^18^. Information can be structured into gradient fields to perform path planning, for example^5^. The accompanied tensor modeling includes higher order dimensions which are used for various tasks in image processing^7,8,22^. Increasing the dimension $\mathbb {R}^{n+1}$ effects the degree of rotation by $[N_\mathrm {{R}} = N(N-1)/2]$ and translation by [$N_\textrm{T} = N$]. In application areas, such as pose estimation^8^ or action recognition^23^, the increased dimension is suitable for the prediction of model-based controls^11,24^ and the general description of multibody systems by trajectory movements^8,22^. Furthermore, approaches in navigation^12^ and control^7^ provide time-optimal trajectory planning that respects the limits of higher-derivative and inverse dynamics to obtain precise forward kinematics^18,22^.

The techniques behind the higher-order description of motion are based on modern differential geometry that contains the Lie group as screw theory^25–27^, Lie algebra^26–28^, and special Euclidean group^19,22,27^ expressed as rigid transformation. Independent rotational Euler angles $\psi ,\theta ,\phi$ in Euclidean space can be defined as the orthogonal group of Lie group $S\mathbb {O}_{3}^{\mathbb {R}}$^26^. The rigid transformation, which is often used to describe the position of multibody systems, also refers to the Lie group and is based on the isometry of Euclidean group $SE_{(n)}$^19,22^. However, motion parameterization of rigid bodies is limited to the definition and properties of suitable orthogonal dual tensors^29^. The boundedness of dynamic solutions and global stability of positive fixed points within these systems can be challenging due to existing inverse problems^30^.

This article extends the sensor perception to temporal aspects and presents an approach to computing and mapping spatio-temporal positions and depth distances. As a contribution, we present the Schlingel diagram as a novel tensor-specific diagram of 4D space and the corresponding cross-dimensional metric. Our approach originates from relativistic image processing^15^, a young field of computer vision that is based on the theory of relativity^31,32^ and the geometric relationships of image processing. This principle extracts 4D information from local sensor and image data and relates it to a spatio-temporal context. This allows the equation-based extraction of sensor and depth maps as well as the detection of superimposed features for the temporal analysis of depth information. We intend to provide new insights in 4D sensor perception in order to simplify the description of 4D positioning and to improve the estimation of depth.

## Preliminaries and notation of relativistic view in 4D space

Relativistic image processing applies sensor and image data to estimate depth distances and positions. The fundamental principle is based on the theory of relativity^31,33–35^, in which the changes in motion of different inertial systems are related to each other and subjected to the geometric properties of space and time^36^. Adapted to sensor information, camera frames can be related to motion-dependent sensor data, considering temporal and physical aspects. This means that inertial changes in position or perspective over time are related to the physical quantities of sensor information. The combination of techniques from computer vision-specific image processing and the differential geometry associated with the theory of relativity allows us to extract temporal-dependent depth information.

We define a space of 4 dimensions that includes time as part of metric space $(\forall$ $\zeta ,x,y,z\in$ $\chi ^{\mu }$). The variable $\zeta [m \cdot s^{-1} \cdot s = m ]$ describes the unit reduced temporal axes of 4D space by involved speed of light of $\{ c=1 \} \in \mathbb {R}^{4}$  ^32^. This allows the verification of temporal change as a meter unit. Whereby, the natural unit ($c=1$) is only valid in 4D space $\mathbb {R}^{4}$. It simplifies the use of relativistic equations and enables the transferability to different sensor types since space and time are measured in the same unit.

The metric of space can be defined as $d: M \times M \xrightarrow {R}$ with the conventional 2-order tensor $\mu : V \times V \xrightarrow {R}$, $\mathbb {R}^{1,4}: (\mathbb {R},\chi ^{\mu })$. In turn, the vector space $V_{\epsilon }(x_\textrm{0})$ is denoted as $V, V_\textrm{1},... , V_\textrm{n}$ with further subvector spaces $U,U_\textrm{1},... ,U_\textrm{n}$. The contained quantity of the environment $\epsilon$ in vector space $V_{\epsilon }(x_\textrm{0})$ $:= \{ x \in \chi ~ | ~ d(x,x_\textrm{0}) < \epsilon \}$ requires a metric, which we define as a metric space ($\chi ,d$) with $x_\textrm{0} \in \chi$ and $\epsilon > 0$. The motion in space is defined as four-vector $\chi ^{\mu }$ and refers to Einstein’s summation convention $(+---)$^31,36^:

1 $$\begin{aligned} d s^{2} = d \zeta ^{2} - d x^{2} - d y^{2} - d z^{2} \hspace{0.0cm} \in \mathbb {R}^{4} \hspace{0.3cm} with \hspace{0.3cm} \zeta ^{2} = c^{2} d t^{2} \end{aligned}$$

Where $\chi ^{\mu } = (\chi ^{0}+\chi ^{1}+\chi ^{2}+\chi ^{3})$ contains the following form:

2 $$\begin{aligned} \chi ^{\mu } = ~A^{\zeta } \vec {e_\mathrm {\zeta }} + A^{x} \vec {e_\textrm{x}} + A^{y} \vec {e_\textrm{y}} + A^{z} \vec {e_\textrm{z}} =~A^{\zeta } \vec {e_\mathrm {\zeta }} + A^{i} =~A^{T} e_\textrm{T} \hspace{0.5cm} \{ \hspace{0.3cm} \chi ^{\mu } = [\zeta , x,y,z] \in \mathbb {R} \end{aligned}$$

Applying the four-vector allows the relativistic view of successive motion. To convert one reference system into another or to compare reference systems with each other, it is necessary to apply the Lorentz transformation^36,37^. The Lorentz transformation relates temporal and spatial coordinates of one reference system to the other. If we compare the motion of position sensors with visual sensors or image-acquired object motions, the relativistic reference can be expressed by Lorentz factor $\Gamma$^32,33,36^:

3 $$\begin{aligned} \Gamma = \dfrac{1}{\sqrt{1-(\dfrac{v}{c})^{2}}} = \dfrac{E_\textrm{kin}}{E_\textrm{0}} + 1 \ge 1 \in \mathbb {R}^{4} \hspace{0.3cm} with \hspace{0.3cm} \{ c^{2} = 1 \} \in \mathbb {R}^{4} \end{aligned}$$

The invariant behavior of Lorentz factor ensures the conversion of different quantities into each other by selective transformation. This contains a temporal contraction, defined as time dilation^31,36^, due to relative shifts between these reference systems. The temporal effect that occurs influences the actual position of spatial coordinates. In relative terms, space and time coordinates change. Velocities between two existing systems can be transformed in form of a boost. This boost shall be expressed as Lorentz-Boost $\tau _{\Gamma }$. The temporal relative view of coordinate systems can be specified as boost matrix along the respective axis of x,y,z:

4 $$\begin{aligned} & X-Direction \left( \begin{array}{c c c c} \Gamma _{0} & -\Gamma _{0} \beta _{0} & 0 & 0 \\ -\Gamma _{0} \beta _{0} & \Gamma _{0} & 0 & 0 \\ 0 & 0 & 1 & 0 \\ 0 & 0& 0& 1 \\ \end{array} \right) \tau _{\Gamma ,x} \end{aligned}$$

5 $$\begin{aligned} & Y-Direction \left( \begin{array}{c c c c} \Gamma _{0} & 0 & -\Gamma _{0} \beta _{0} & 0 \\ 0 & 1 & 0 & 0 \\ -\Gamma _{0} \beta _{0} & 0 & \Gamma _{0}& 0 \\ 0 & 0 & 0 & 1 \\ \end{array} \right) \tau _{\Gamma ,y} \end{aligned}$$

6 $$\begin{aligned} & Z-Direction \left( \begin{array}{c c c c} \Gamma _{0} & 0 & 0 & -\Gamma _{0} \beta _{0} \\ 0 & 1 & 0 & 0 \\ 0 & 0 & 1 & 0 \\ -\Gamma _{0} \beta _{0} & 0 & 0 & \Gamma _{0} \\ \end{array} \right) \tau _{\Gamma ,z} \end{aligned}$$

7 $$\begin{aligned} & with~ \beta _\textrm{0} = \dfrac{v}{c} \hspace{0.3cm} and \hspace{0.3cm} \{ c^{2} = 1 \} \in \mathbb {R}^{4} \end{aligned}$$

The boost matrix describes a Lorentz transformation which differentiates between rotations as well as reversible [t $\xrightarrow -t$] and parity [(x,y,z) $\xrightarrow (-x,-y,-z)$] transformations. It describes the change in velocity along a specified spatial direction. By applying the Lorentz transformation, estimated position coordinates (*R*|*T*) and measured times can be related from one inertial reference system to another moving reference system. This inertial change of coordinates is expressed by Lorentz boost. We describe the resulting boost matrix in time $\tau _{\Gamma }$ by the following:

8 $$\begin{aligned} \tau _{\Gamma } = \left( \begin{array}{c c c c} \Gamma & -\Gamma \dfrac{v_\textrm{x}}{c} & -\Gamma \dfrac{v_\textrm{y}}{c} & - \Gamma \dfrac{v_\textrm{z}}{c} \\ & & & \\ -\Gamma \dfrac{v_\textrm{x}}{c} & 1 + (\Gamma - 1) \dfrac{v_\textrm{x}^{2}}{v^{2}} & (\Gamma - 1) \dfrac{v_\textrm{x} v_\textrm{y}}{{v}^{2}} & (\Gamma - 1) \dfrac{v_\textrm{x} v_\textrm{z}}{{v}^{2}} \\ & & & \\ -\Gamma \dfrac{v_\textrm{y}}{c} & (\Gamma - 1) \dfrac{v_\textrm{y} v_\textrm{x}}{{v}^{2}} & 1 + (\Gamma - 1) \dfrac{v_\textrm{y}^{2}}{v^{2}} & (\Gamma - 1) \dfrac{v_\textrm{y} v_\textrm{z}}{{v}^{2}}\\ & & & \\ - \Gamma \dfrac{v_\textrm{z}}{c} & (\Gamma - 1) \dfrac{v_\textrm{z} v_\textrm{x}}{{v}^{2}} & (\Gamma - 1) \dfrac{v_\textrm{z} v_\textrm{y}}{{v}^{2}} & 1 + (\Gamma - 1) \dfrac{v_\textrm{z}^{2}}{v^{2}} \\ \end{array} \right) \hspace{0.5cm} with~ v = \sqrt{v_\textrm{x}^{2} + v_\textrm{y}^{2} + v_\textrm{z}^{2}} \end{aligned}$$

The Jacobian matrix of the Lorentz transformation contains the four-vector^31^ of the special theory of relativity which depends on the magnitude of velocity $v_\textrm{x}, v_\textrm{y}, v_\textrm{z}$. It represents the partial derivatives of the transformed coordinates with respect to the original coordinates^38^. The special Lorentz transformation^39,40^ defines transformations in parallel moving systems through the Lorentz group $O_{(3,1)}$, where the coordinate axes do not have a relative rotation to the system at rest.

9 $$\begin{aligned} & \Lambda _\textrm{s} = \left( \begin{array}{c c } cosh(\chi ) & sinh(\chi ) \\ sinh(\chi ) & cosh(\chi ) \end{array} \right) \hspace{1cm} \Bigg \{ \begin{array}{l} sinh_{(\chi )}: \langle - \infty< f_\mathrm {(x)}< \infty \rangle \\ cosh_{(\chi )}: \langle - 1 \le f_\mathrm {(x)} < \infty \rangle \end{array} \end{aligned}$$

10 $$\begin{aligned} & cosh(\chi ) = \Gamma \hspace{0.3cm} and \hspace{0.3cm} sinh(\chi ) = \Gamma \cdot \dfrac{v}{c} = \Gamma \beta \end{aligned}$$

## Schlingel diagram: tensor-based diagram of 4D space

The Schlingel diagram defines a novel approach of coordinate system in which space- and time-dependent information are expressed by 10 different degrees of freedom (DoF), consisting of 4 translations and 6 rotations^15^. The novel diagram considers the temporal aspects of space and extends the view of Minkowski diagram^33,40^ and mathematical subspaces, in which higher-dimensional information are represented as spatio-temporal coordinates.

**Fig. 2:**
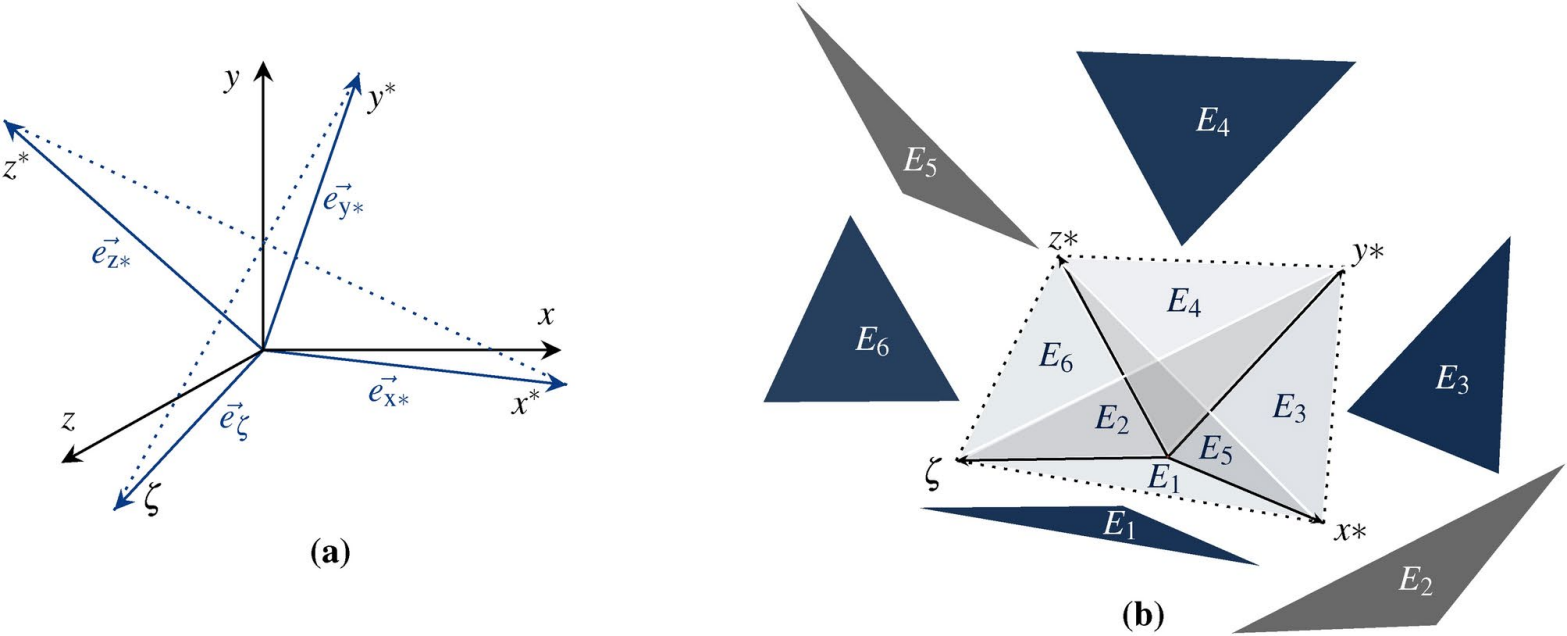
The illustration shows the novel approach of a 4D diagram that converts Euclidean coordinates into spatio-temporal coordinates by composing tensor bases, shown in (**a**). The tensor bases of the Schlingel diagram constitute the intersection and act as a linearly independent generation system of different information between $\mathbb {R}^{3}$ and $\mathbb {R}^{4}$. The spatio-temporal motion in the tensor-based diagram can be located between 6 planes, as shown in (**b**).

We describe an extended dual space $V^{*} = \{f: V \rightharpoondown \chi |~where~f~is~linear\}$ with spatio-temporal dimension. The covectors $e^{\nu }=\{ e_{x},e_{y},e_{z} \}$ of vector space *V* are extended to the bases $e_{*}=\{ e_{\zeta },e_{x{*}},e_{y{*}},e_{z{*}} \}$ of dual space $V^{*}$ defined according Kronecker delta $\{ e^{\nu }(e_{*}) = \delta _{*}^{\nu } \}$. In this context, $\vec {e}_{\zeta },\vec {e}_{x^{*}},\vec {e}_{y^{*}}$ and $\vec {e}_{z^{*}}$ form the linear mappings of their corresponding vectors *x*, *y*, *z*. The result of the base expansion replicates a dual space $V^{*}$ from the original Euclidean coordinate system, which is defined as a Schlingel diagram, as shown in Fig. 2.

The tensor-based diagram, shown in Fig. 2, offers the possibility of relating not only local but also temporal dependencies of information. The planes $E_{1},~...,~E_{6}$ shall be spanned as a part of vector space *V* over the field $\mathbb {F}$ as $\{ E \subset V \} \in \mathbb {F}:$

11 $$\begin{aligned} & E_{1} = span\{x^{*},\zeta \} = \{ x^{*} e_{x*} + \zeta e_{\zeta }~|~e_{x*},e_{\zeta } \in \mathbb {F} \} \end{aligned}$$

12 $$\begin{aligned} & E_{2} = span\{y^{*},\zeta \} = \{ y^{*} e_{y*} + \zeta e_{\zeta }~|~e_{y*},e_{\zeta } \in \mathbb {F} \} \end{aligned}$$

13 $$\begin{aligned} & E_{3} = span\{x^{*},y^{*} \} = \{ x^{*} e_{x*} + y^{*} e_{y*} ~|~ e_{x*},e_{y*} \in \mathbb {F} \} \end{aligned}$$

14 $$\begin{aligned} & E_{4} = span\{y^{*},z^{*} \} = \{ y^{*} e_{y*} + z^{*} e_{z*} ~|~ e_{y*},e_{z*} \in \mathbb {F} \} \end{aligned}$$

15 $$\begin{aligned} & E_{5} = span\{x^{*},z^{*} \} = \{ x^{*} e_{x*} + z^{*} e_{z*} ~|~ e_{x*},e_{z*} \in \mathbb {F} \} \end{aligned}$$

16 $$\begin{aligned} & E_{6} = span\{z^{*},\zeta \} = \{ z^{*} e_{z*} + \zeta e_{\zeta } ~|~ e_{z*},e_{\zeta } \in \mathbb {F} \} \end{aligned}$$

$x^{*},y^{*},z^{*},\zeta$ forms the linear combination, with the scalar coefficients $e_{x*},e_{y*},e_{z*}$ and $e_{\zeta }$. Information can be described by 6 different planes of temporal ($E_{1},E_{2},E_{6}$) and non-temporal ($E_{3},E_{4},E_{5}$) dependencies.

**Fig. 3:**
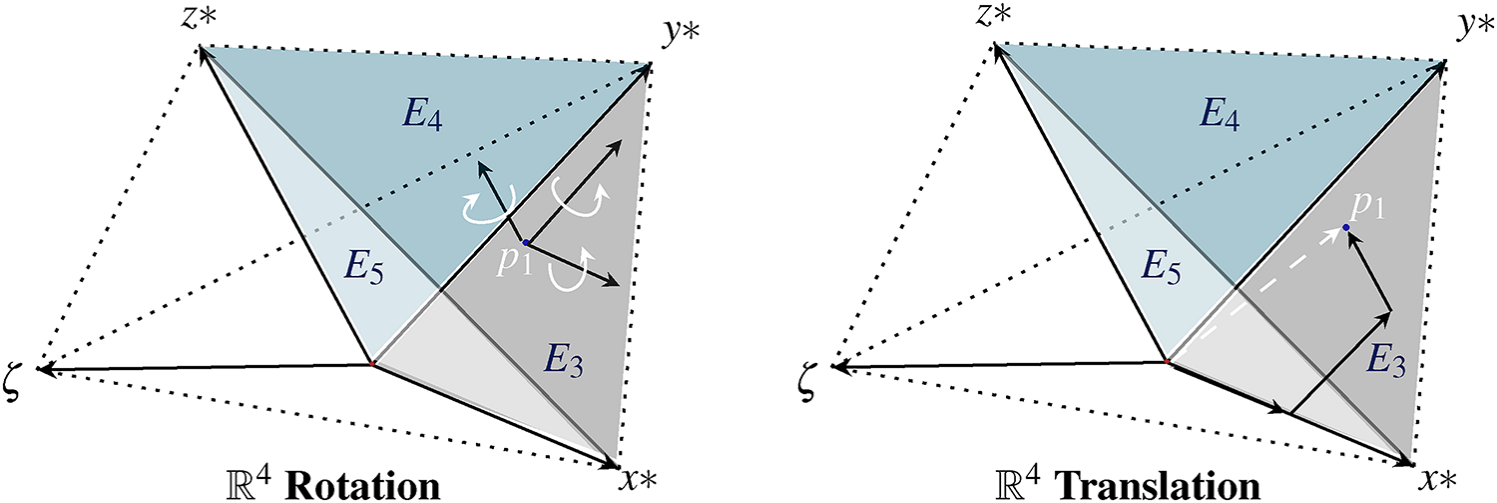
The Schlingel diagram rebuilds the Euclidean diagram by the planes $E_{3}, E_{4}$ and $E_{5}$. If no temporal aspects are considered, the measuring point $p_{1}$ is located within the Euclidean planes. Positions are described in the case of $\{ \zeta =0 \}$ by 3 rotations and 3 translations. A temporal shift causes the positional shift to the other planes. Under relativistic aspects, the resulting position will be described by 6 rotations and 4 translations.

Further, the information inside the plane of Schlingel diagram $\mathbb {R}^{4}$ can be transformed to Euclidean space $\mathbb {R}^{3}$ by linear mapping since the bases of Euclidean space are linear dependent to the bases of Schlingels dual space^38^. In this context, the Euclidean coordinate system can be mapped as a non-temporal part of the Schlingel diagram, shown in Fig. 3.

The state of measuring points can be localized by different planes. At rest, the measuring points are located between the planes $E_{3}, E_{4}$ and $E_{5}$. If we relate the time between the systems, the measuring point shifts along the $\zeta$ axis. In the Euclidean diagram, the vectors *x*, *y*, *z* would appear smaller, as the temporal shift would not be detectable. In the Schlingel diagram, the measuring point is no longer described by 3 planes instead it is defined by 6 planes. Besides the position of measuring points, we describe the inverse behavior of spatial coordinates, which changes the arrangement of planes in the Schlingel diagram. Various states can be defined in which the geometry of the diagram changes depending on the spatial direction of spread. This enables the asynchronous processing of information that affects negative times, position changes and the matching of different information due to relativistic considerations.

**Fig. 4:**
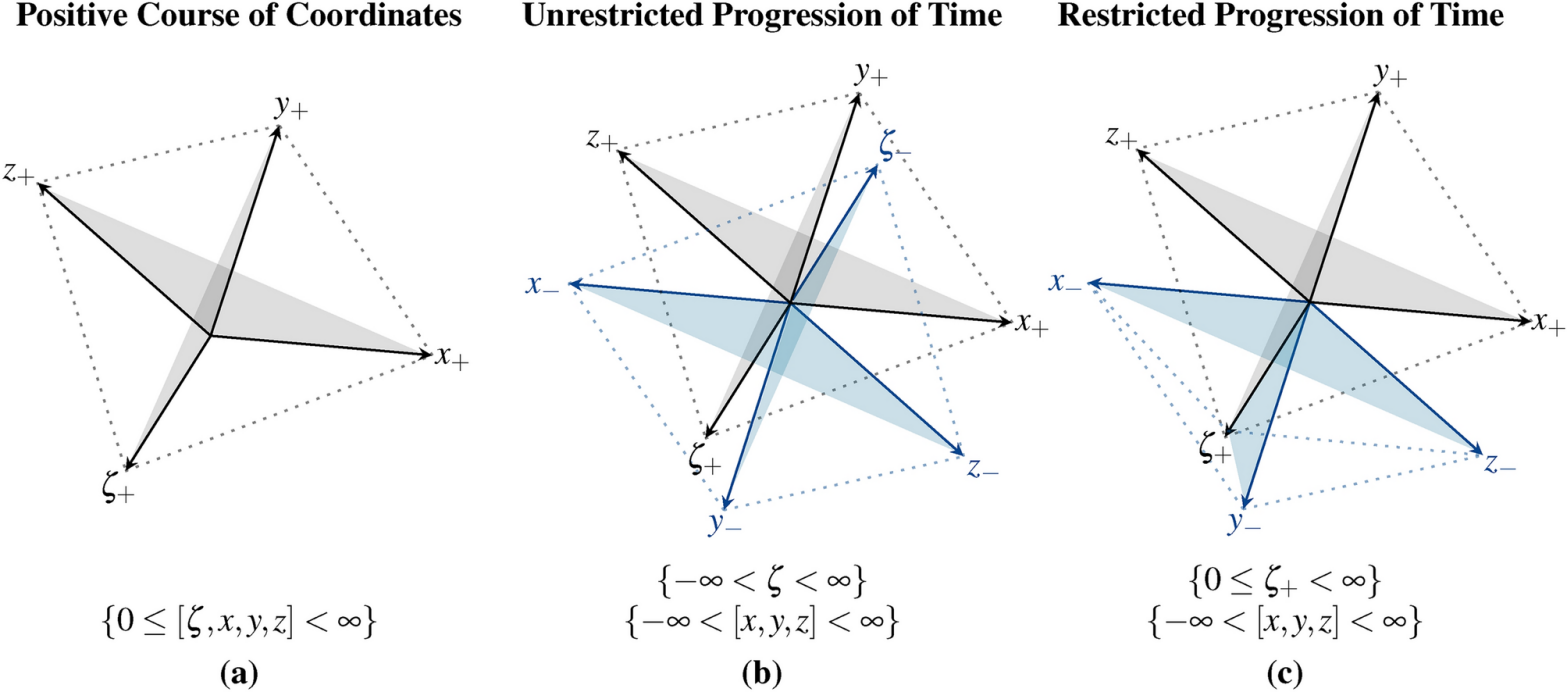
The illustration introduces the inverse behavior of Schlingel coordinates. The orientation of the information along the spatial axes in $\{ p_{1} \rightarrow -\infty \}$ or $\{p_{1} \rightarrow \infty \}$ changes the position of planes in the diagram. In (**a**), the Schlingel coordinates follow a positive course. The coordinates in (**b**) are located between $- \infty$ and $\infty$. In (**c**), the temporal coordinate can only assume positive values.

Fig. 4 introduces three inverse forms in the behavior of Schlingel coordinates. In (a), information has a positive course along the spatial axes. Magnitude functions can be described within the 6 planes of positive diagram course. The cases (b) and (c) extend the Schlingel diagram by 6 further planes. (b) describes the unrestricted progression of spatial and temporal coordinates. This means that information can have a positive and negative trend along their spatial and temporal axes towards the relativistic reference. In (c), only the temporal axis has a positive progression. The Euclidean coordinates can trend along positive or negative axis.

## Spatio-temporal motion by sensor information

**Fig. 5:**
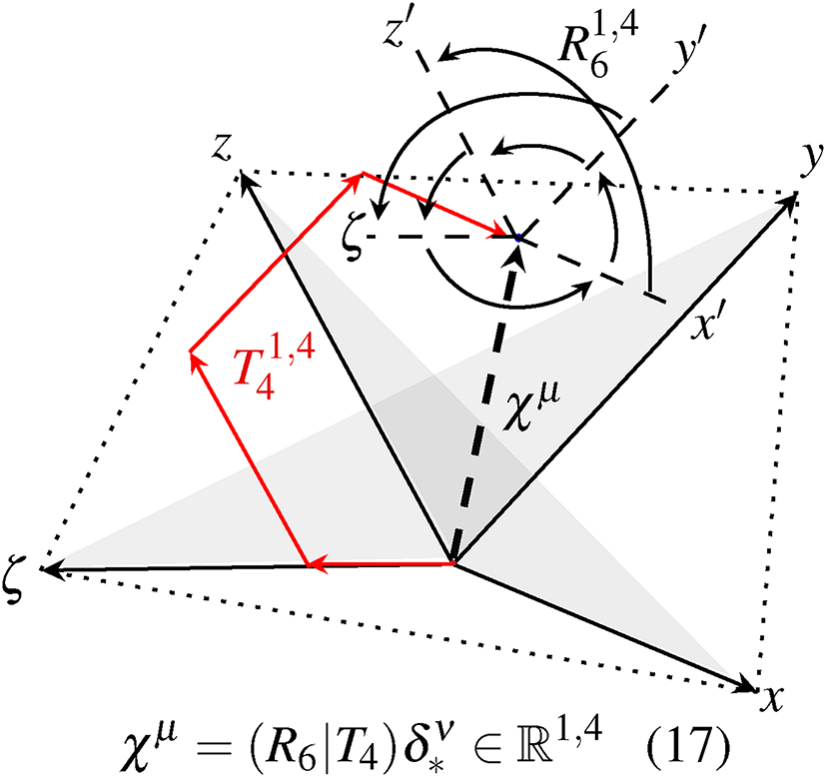
The illustration shows the motion of 10 DoF using the Schlingel diagram. The DoF comprises 4 translations $T_{4}^{1,4}$ and 6 rotations $R_{6}^{1,4}$ in which the spatio-temporal measuring points are indicated on 6 planes, formed by 4 different axes. The resulting motion can be expressed as $\chi ^{\mu }$.

The temporal part of 4D space offers the possibility to expand the sensor perception. Physical changes in an environment, which can be measured by different types of sensors, are referenced via space and time. The sensors can form a measurable relationship to each other, which we describe as relativity. If we refer to a moving sensor and apply the inertial system to it, the surrounding sensors behave relative to the moved inertial system. The temporal aspects, in which the sensor’s own motion behaves relative to the environmental changes, can be related to various sensor information. The description of motion also changes under these temporal aspects. The 4D space is described by 10 DoF based on the principles of the theory of relativity. The Schlingel diagram in Fig. 5 allows us to visualize the different DoFs of 4D space.

**Fig. 6:**
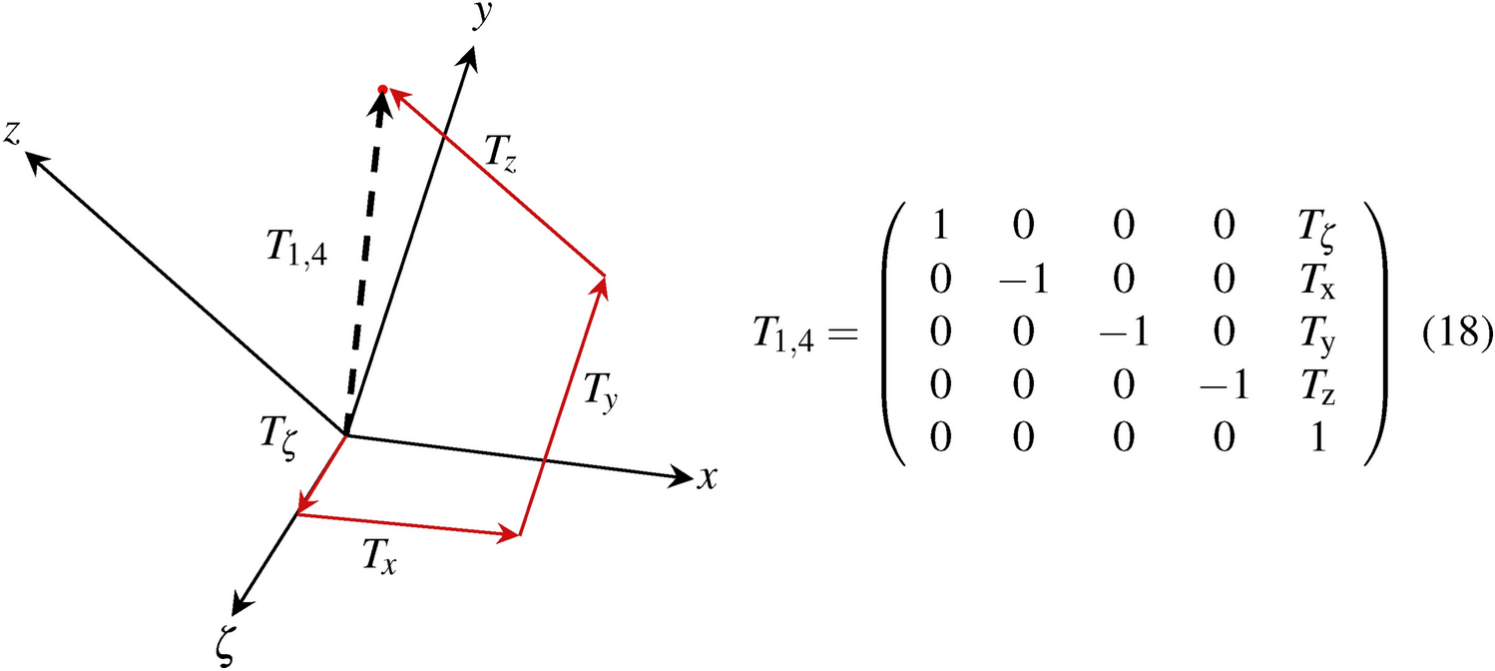
The different components of translation can be illustrated in 4D space using the Schlingel diagram. The resulting translation ${T_{1,4}} \in \mathbb {R}^{4}$ consists of the Euclidean components $T_{x},T_{y},T_{z}$ and the temporal component $T_{\zeta }$.

The resulting variable $\chi ^{\mu }$ defines the four-vector and contains 10 DoF’s in 4D space consisting of 4 translations [$T_{\zeta },T_{x},T_{y},T_{z}$] and 6 rotations [${R}_{\zeta , x},{R}_{\zeta ,y},{R}_{\zeta ,z},{R}_{x,y},{R}_{x,z},{R}_{y,z}$]. We generalize the orthogonal group $O_{(3)}$ to the Lorentz group $O_{(3,1)}$^27^ in order to describe boosts and rotations. Further, we use the Poincaré group^27,41^, which includes not only the Lorentz transformations but also translations to transfer space symmetric coordinates. This allows the completion of spacetime symmetries. The homogeneous matrix (Eq. 18) is subjected to Einstein’s sum convention (Eq. 1) and illustrated in Fig. 6.

The temporal translation $T_{\zeta }$, as shown in Fig. 6, reflects temporal influences like time dilation or Lorentz contraction that occur when different inertial frames are related to each other. The resulting translation affects the geometry of space and their relative positions.

**Fig. 7:**
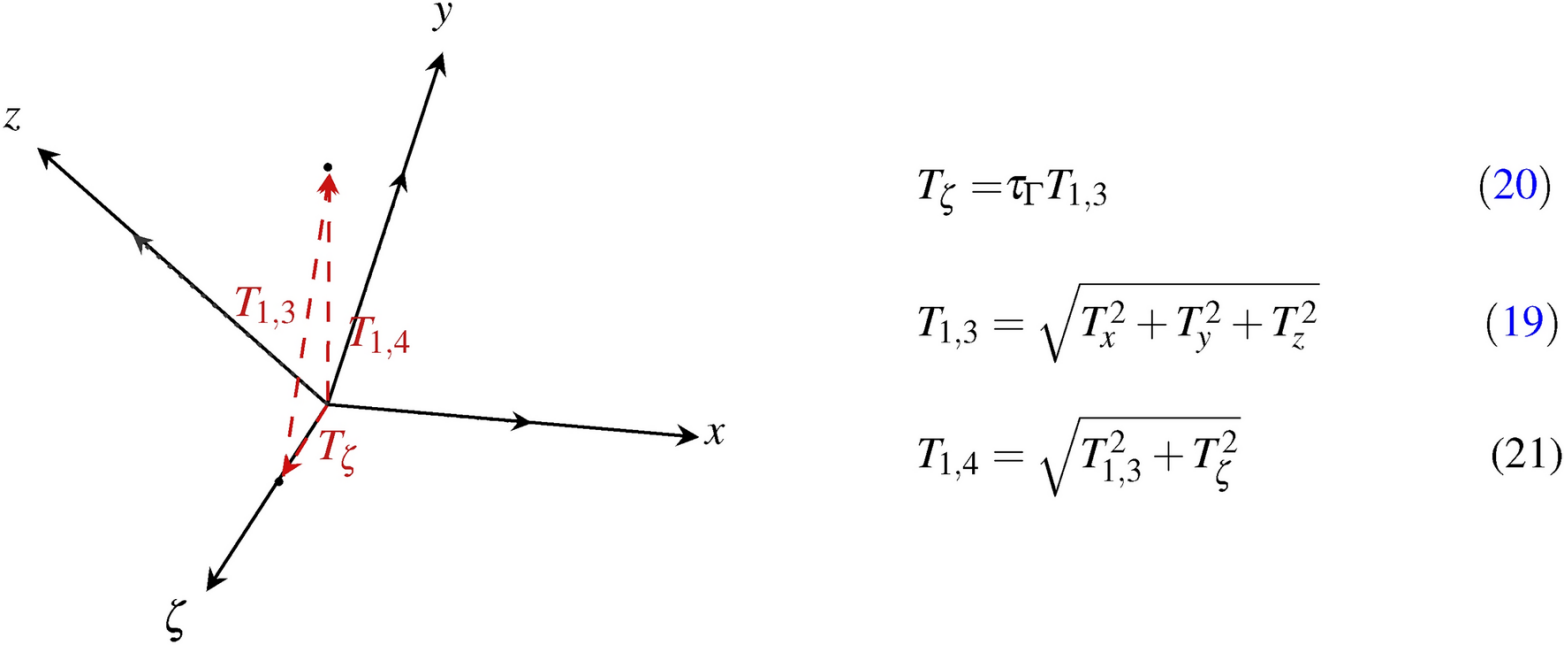
The illustration shows the resulting components of 3rd and 4th-dimensional translation combined in the Schlingel diagram. The 4D translation ${T}_{1,4}$ is vectorially composed of the Euclidean ${T}_{1,3}$ and temporal translation ${T}_{\zeta }$.

The components $T_{x},T_{y},T_{z}$ correspond to the translational displacement of Euclidean space, summarized as ${T}_{1,3}$ in the Schlingel diagram of Fig. 7. Thereby, the resulting magnitude of ${T}_{1,3}$ follows from Eq. 18.

19 $$\begin{aligned} {T}_\textrm{1,4} = \left( \begin{array}{c c c} 1 & 0 & T_{\zeta }\\ 0 & -1 & T_\textrm{1,3}\\ 0 & 0 & 1 \\ \end{array} \right) \hspace{0.4cm} with \hspace{0.4cm} {T}_{1,3} = \sqrt{T_{x}^{2} + T_{y}^{2} + T_{z}^{2}} \end{aligned}$$

We assume that an initial temporal change of $(x,y,z) \rightarrow (x',y',z') = (x+z,y+z,x+y)$ with $t \ge 0$ is consistent in all spatial directions and that directional kinematic changes are subjected to the physical laws of equivalence principle^42^. The linear transformed Lorentz boost shall be applied to describe the spatio-temporal displacement along $\zeta$-direction. To verify our assumption, we consider the Poincaré transformation $\{ \Lambda _{\nu }^{\mu } T^{\nu } + \chi ^{\mu } \}$ which combines the actual Lorentz transformation $\Lambda$ with a spatial and temporal translation as a four-vector $\chi$. In this context, the temporal translation $T_{\zeta }$ is to be defined by $\tau _{\Gamma }$ and ${T}_{1,3}$ (Eq. 20).

20 $$\begin{aligned} \Lambda _{\nu }^{\mu } T^{\nu } \in V \rightarrow {T}_{\zeta } \in V^{*} := \tau _{\Gamma } {T}_{1,3} \end{aligned}$$

We compose the 4D translation ${T}_{1,4}$ from the resulting Euclidean translation ${T}_{1,3}$ and the temporal translation $T_{\zeta }$. The addition of all translation components shall be illustrated subsequently in the Schlingel diagram of Fig. 7.

The resulting magnitude of $T_{1,3}$ is formed by the vector variables $T_{x}, T_{y}$ and $T_{z}$, shown in Fig. 7. The time-related translation $T_{\zeta }$ describes the temporal influence from one inertial system to another moving inertial system. For this reason, we relate the Lorentz boost $\tau _{\Gamma }$ to the resulting translation quantities of Euclidean components $T_{1,3}$. In order to determine the transformation parameter between $\mathbb {R}^{3}$ and $\mathbb {R}^{4}$, we substitute Eq. 20 into Eq. 21.

$$\begin{aligned} {T}_{1,4} = \sqrt{ T_{1,3}^{2} + T_{1,3}^{2} \cdot \tau _{\Gamma }^{2}} \hspace{1.5cm} \text {(Eq.~20) in (Eq.~21)} \end{aligned}$$

22 $$\begin{aligned} {T}_{1,4}^{2} = T_{1,3}^{2} {{(1+\tau _{\Gamma }^{2})} } \end{aligned}$$

**Fig. 8:**
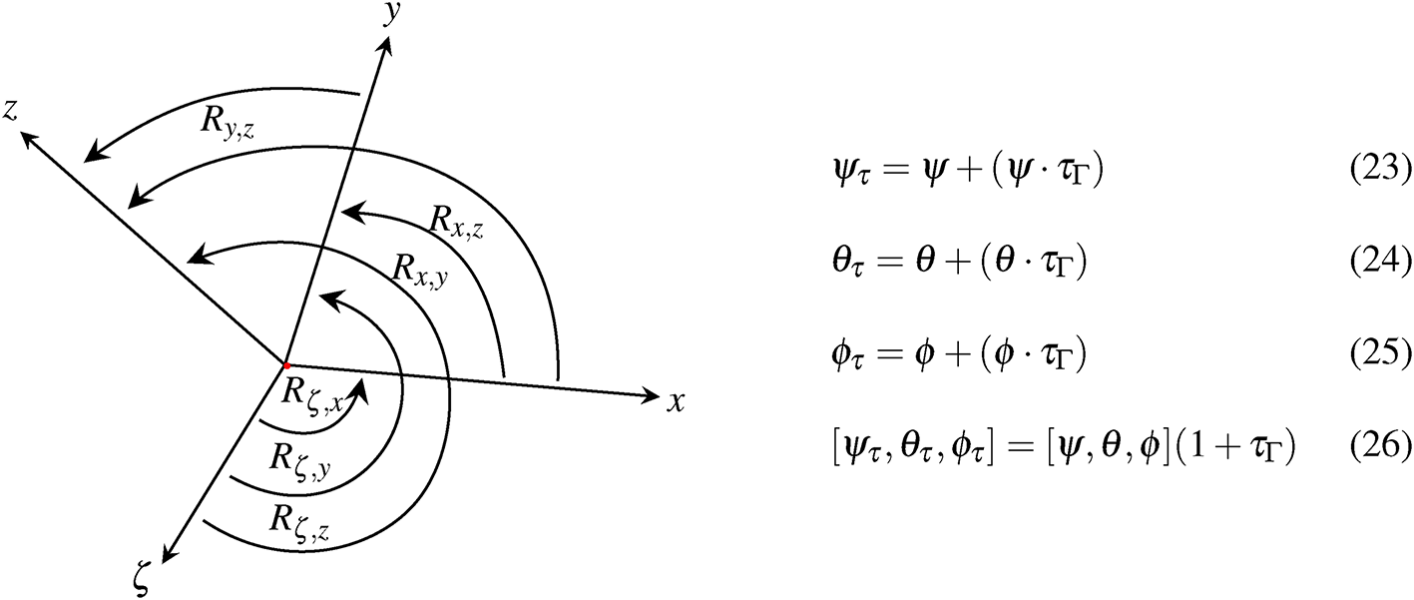
The illustration shows the different types of 4D rotation. The 4D rotation contains 3 time-dependent $\psi _{\tau },\theta _{\tau },\phi _{\tau }$ and time-independent angles $\psi ,\theta ,\phi$. The time-dependent angles contain an additional phase shift due to the Lorentz boost which we express as $(1+\tau _{\Gamma })$.

We extract the temporal factor of reverse and forward transformation between $\mathbb {R}^{3}$ and $\mathbb {R}^{4}$ from Eq. 22:
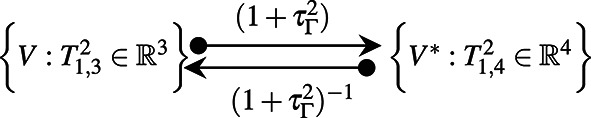


In addition to the translation, we define the 4D orientation by different types of rotational motion. The angular displacements are divided into 3 time-dependent $\phi _{\tau }, \theta _{\tau }, \psi _{\tau }$ and time-independent components $\phi , \theta , \psi$, resulting in a total of 6 rotations, as shown in Fig. 8.

The respective rotations of Fig. 8 can be described as follows.

27 $$\begin{aligned} R_\textrm{x,y} & \hookrightarrow R_\textrm{z}(\psi ) : \left( \begin{array}{c c c c} cos\psi & -sin\psi & 0 & 0\\ sin\psi & cos\psi & 0 & 0\\ 0 & 0 & 1 & 0\\ 0 & 0 & 0 & 1 \\ \end{array} \right)  &~ R_\textrm{x,z} \hookrightarrow R_\textrm{y}(\theta ): \left( \begin{array}{c c c c} cos\theta & 0 & sin\theta & 0\\ 0 & 1 & 0 & 0\\ -sin\theta & 0 & cos\theta & 0\\ 0 & 0 & 0 & 1 \\ \end{array} \right)  \\ R_\textrm{y,z} &\hookrightarrow R_\textrm{x}(\phi ): \left( \begin{array}{c c c c} 1 & 0 & 0& 0\\ 0 & cos\phi & -sin\phi & 0\\ 0 & sin\phi & cos\phi & 0\\ 0 & 0 & 0 & 1 \\ \end{array} \right) &~ R_\mathrm {\zeta ,z}  \hookrightarrow R_\textrm{z}(\psi _{\tau }): \left( \begin{array}{c c c c} 1 & 0 & 0 & 0\\ 0 & 1 & 0 & 0\\ 0 & 0 & cos\psi _{\tau } & sin\psi _{\tau }\\ 0 & 0 & -sin\psi _{\tau } & cos\psi _{\tau } \end{array} \right) \\ R_\mathrm {\zeta ,y}  & \hookrightarrow R_\textrm{y}(\theta _{\tau }): \left( \begin{array}{c c c c} 1 & 0 & 0 & 0\\ 0 & cos\theta _{\tau } & 0 & sin\theta _{\tau }\\ 0 & 0 & 1 & 0\\ 0 & -sin\theta _{\tau } & 0 & cos\theta _{\tau } \\ \end{array} \right)  ~&R_\mathrm {\zeta ,x} \hookrightarrow R_\textrm{x}(\phi _{\tau }): \left( \begin{array}{c c c c} cos\phi _{\tau } & 0 & 0 & -sin\phi _{\tau }\\ 0 & 1 & 0 & 0\\ 0 & 0 & 1 & 0\\ sin\phi _{\tau } & 0 & 0 & cos\phi _{\tau } \\ \end{array} \right)   \end{aligned}$$

We summarize the rotations of Eq. 27 as Eq. 28 and simplify it to Eq. 29.

28 $$\begin{aligned} R_\textrm{1,4} = R_\mathrm {z(\psi )} R_\mathrm {y(\theta )} R_\mathrm {x(\phi )} R_\mathrm {z(\psi _{\tau })} R_\mathrm {y(\theta _{\tau })} R_\mathrm {x(\phi _{\tau })} \end{aligned}$$

The Eq. 23, Eq. 24 and Eq. 25 can be substituted into Eq. 28.

$$\begin{aligned} R_{1,4} =&R_{z(\psi )} R_{y(\theta )} R_{x(\phi )} R_{z(\psi + \psi \tau _{\Gamma })} R_{y(\theta + \theta \tau _{\Gamma })} R_{x(\phi + \phi \tau _{\Gamma })} \\ \end{aligned}$$

The spatio-temporal rotation can be summerized by $R_{\alpha +\beta } = R_{\alpha } R_{\beta }$ and $R_{\alpha } R_{\alpha } = R_{\alpha }^{2}$ for $\{ \psi ,\theta ,\phi \} = [0 \le 2 \pi ].$

$$\begin{aligned} R_{1,4} =&R_{z(\psi )} R_{y(\theta )} R_{x(\phi )} R_{z(\psi )} R_{y(\theta )} R_{x(\phi )} R_{z(\psi \tau _{\Gamma })} R_{y( \theta \tau _{\Gamma })} R_{x(\phi \tau _{\Gamma })}\\ R_{1,4} =&R_{z(\psi )}^{2} R_{y(\theta )}^{2} R_{x(\phi )}^{2} R_{z(\psi \tau _{\Gamma })} R_{y(\theta \tau _{\Gamma })} R_{(\phi \tau _{\Gamma })} \end{aligned}$$

Eq. 29 results from $R_{1, 3} = R_{z(\psi )} R_{y(\theta )} R_{x(\phi )} \in \mathbb {R}^{3}$ and $R_{\zeta } = R_{z(\psi \tau _{\Gamma })} R_{y(\theta \tau _{\Gamma })} R_{x(\phi \tau _{\Gamma })}$.

$$\begin{aligned} R_{1,4} = R_{1,3}^{2} R_{z(\psi \tau _{\Gamma })} R_{y(\theta \tau _{\Gamma })} R_{x(\phi \tau _{\Gamma })} \end{aligned}$$

29 $$\begin{aligned} R_{1,4} = R_{1,3}^{2} R_{\zeta } \end{aligned}$$

In case of small angles, the temporal rotations can simplified by first-order Taylor expansion.

30 $$\begin{aligned} & R_{x}(\phi + \phi \tau _{\Gamma }) \approx R_{x}(\phi ) + \phi \tau _{\Gamma } \dfrac{\partial R_{x}(\phi )}{\partial \phi } \end{aligned}$$

31 $$\begin{aligned} & R_{y}(\theta + \theta \tau _{\Gamma }) \approx R_{y}(\theta ) + \theta \tau _{\Gamma } \dfrac{\partial R_{y}(\theta )}{\partial \theta } \end{aligned}$$

32 $$\begin{aligned} & R_{z}(\psi + \psi \tau _{\Gamma }) \approx R_{z}(\psi ) + \psi \tau _{\Gamma } \dfrac{\partial R_{z}(\psi )}{\partial \psi } \end{aligned}$$

The introduced derivations of Eq. 22 and Eq. 29 can be used to extend the motion-related four-vector $\chi ^{\mu }$ by associated DoFs.

33 $$\begin{aligned} \chi ^{\mu } = A^{T} e_\textrm{T} = ({R}^{1,4}_{6}|{T}^{1,4}_{4}) e_\textrm{T} = (R_{1,3}^{2} R_{ \zeta }| T_{1,3}(1+\tau _{\Gamma }^{2}) ) \delta _{*}^{\nu } \in \mathbb {R}^{1,4} \end{aligned}$$

The DoF-extended four-vector contains the temporal translation of $(1+\tau _{\Gamma }^{2})$. The temporal rotation $R_{\zeta }$ comprises $R_{z(\psi \tau _{\Gamma })} R_{y(\theta \tau _{\gamma })}$ and $R_{x(\phi \tau _{\Gamma })}$. The spatial DoFs are expressed as $T_{1,3}$ and $R_{1,3}^{2}$.

## Cross-dimensional metric

This section introduces a metric to incorporate cross-dimensional information into the calculation of missing quantities. In terms of relative image processing, 2D images can be related to 3D sensor data to estimate the missing depth of a 2D image.

Previously, we described that the number of rotations and translations changes with increasing dimensionality, as shown in Eq. 34. Thereby, the translation increases linearly with each additional dimension *N* $(N_{T} = N )$. Conversely, the number of rotations increases non-linearly with further dimension, similar to a second-order polynomial $(N_{R} = 0.5~N^{2}-0.5~N )$. In this context, the dimension-related change of DoF appears as follows.

34 $$\begin{aligned} & \mathbb {R}^{N}: ([N_\mathrm {{R}} = N(N-1)/2] | [N_\textrm{T} = N] ) = ( [N_{R} = 0.5~N^{2}-0.5~N ]| [N_\textrm{T} = N] ) \end{aligned}$$

35 $$\begin{aligned} & \mathbb {R}^{1}: ({R}^{1,1}_{0}|{T}^{1,1}_{1}) \rightarrow \mathbb {R}^{2}: ({R}^{1,2}_{1}|{T}^{1,2}_{2}) \rightarrow \mathbb {R}^{3}:({R}^{1,3}_{3}|{T}^{1,3}_{3}) \rightarrow \mathbb {R}^{4}:({R}^{1,4}_{6}|{T}^{1,4}_{4}) \end{aligned}$$

36 $$\begin{aligned} & \mathbb {R}^{1}:({R}^{1,1}_{0}|{T}^{1,1}_{1}) \rightarrow \mathbb {R}^{2}: ({R}^{1,2}_{1}|{T}^{1,2}_{2}) \rightarrow \mathbb {R}^{3}:({R}^{1,3}_{3}|{T}^{1,3}_{3}) \rightarrow \mathbb {R}^{4}:(R_{1,3}^{2} R_{ \zeta }| T_{1,3}(1+\tau _{\Gamma }^{2}) ) \delta _{*}^{\nu } \end{aligned}$$

We assume that the quantities of preferred direction $$(R_{xy}$$ and $$T_{x},T_{y})$$ are known in $$\mathbb {R}^{2}$$. In general, these quantities also exist in $$\mathbb {R}^{3}$$ and $$\mathbb {R}^{4}$$. If the remaining variables of $$\mathbb {R}^{3}$$ ($$R_{xz},R_{yz}$$ and $$T_{z}$$) and $$\mathbb {R}^{4}$$ ($$R_{\zeta x},R_{\zeta y},R_{\zeta z}, R_{x z},R_{yz}$$ and $$T_{\zeta },T_{z}$$) are equal to 0, the resulting translations and rotations of the different dimensions are identical. Conversely, the higher dimensions can be used to infer unknown quantities of the other preferred directions such as $$(R_{xz}$$ and $$T_{x},T_{z})$$ or $$(R_{zy}$$ and $$T_{z},T_{y})$$.

**Fig. 9:**
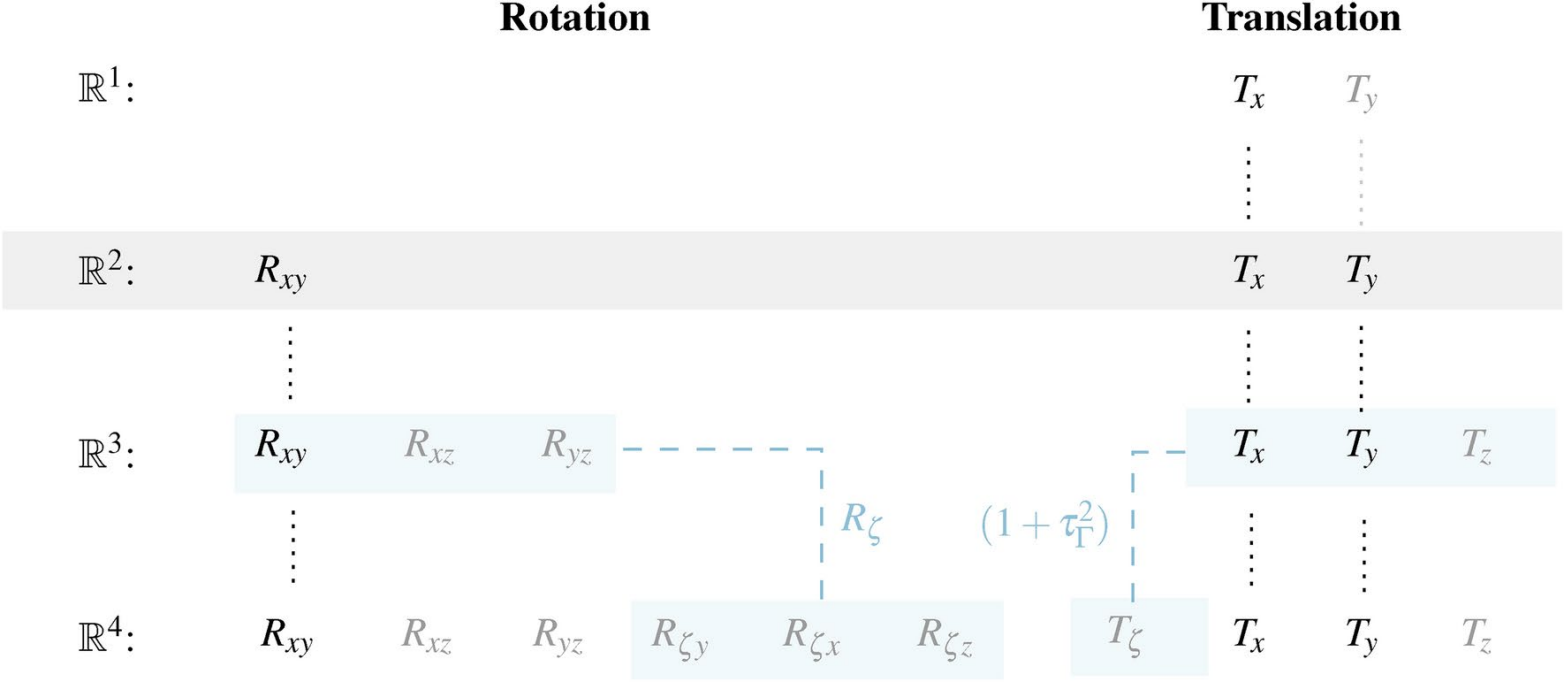
The illustration describes the cross-dimensional relationship between the degrees of rotation and translation departing from $\mathbb {R}^{2}$. The gray-colored sizes refer to existing components of higher dimensions which expands the vision and affects the result of values.

Fig. 9 describes an extensive correlation between different dimensions in terms of DoF. For example, time-related rotation of fourth dimension $R_{\zeta x y},~R_{\zeta x z},~R_{\zeta yz}$ can already influence the sizes of the third dimension. As a result, missing information, like $T_{y}~\in ~\mathbb {R}^{1}$ or non-existing rotations in $\mathbb {R}^{1}$, can be extracted by using the next higher dimensions (like $T_{x}~and~T_{y}~\in ~\mathbb {R}^{2}$) when we process information as a cross-dimensional system of equations.

**Fig. 10:**
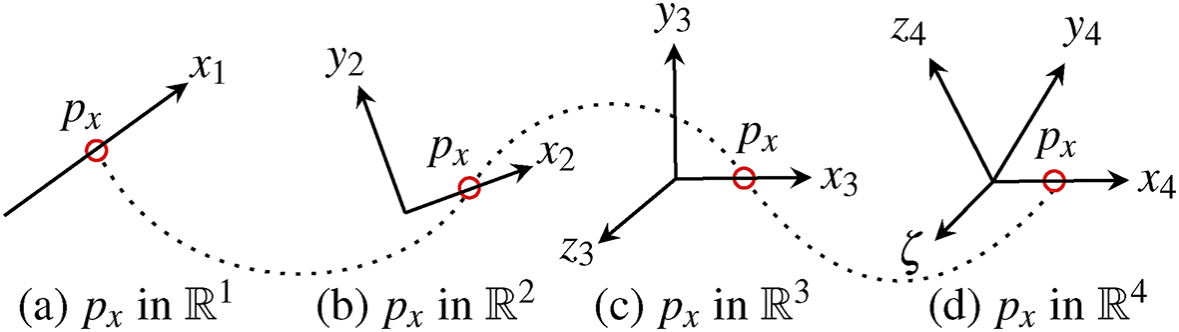
The illustration describes a quantity $p_{x}$, which is determined from various dimensions of the preferred x-direction.

A cross-dimensional system of equations offers the possibility to determine unknown quantities using different sensor information from an environment which are physically related to each other. We transfer the example of the preferred directions $\{ \mathbb {R}^{1}: x \}$ and $\{ \mathbb {R}^{2}: x, y \}$ from Fig. 9 in different spatial directions. The result is illustrated in Fig. 10.

The associated dimensions in Fig. 10 can be related to the x-direction, including the rules of Euclidean and higher-order Minkowski space with the metric $\eta _{\mu \nu }:=\langle +--- \rangle \in \mathbb {R}^{1,4}$. Under these conventions, the cross-dimensional system of equations for x can be described as follows.

37 $$\begin{aligned} ~ for~ \left[ \begin{array}{c} \mathbb {R}^{1,1} \\ \mathbb {R}^{1,2} \\ \mathbb {R}^{1,3} \\ \mathbb {R}^{1,4} \\ \end{array} \right] = \left[ \begin{array}{r r r r} 0 & 1 & 0 & 0 \\ 0 & 1 & 1 & 0 \\ 0 & 1 & 1 & 1 \\ 1 & -1 & -1 & -1 \end{array} \right] \hspace{0.3cm} and \hspace{0.3cm} \left[ \begin{array}{r} a_{1,1} 0 + a_{1,2} x + a_{1,3} 0 + a_{1,4} 0\\ a_{2,1} 0 + a_{2,2} x + a_{2,3} y + a_{2,4} 0\\ a_{3,1} 0 + a_{3,2} x + a_{3,3} y + a_{3,4} z\\ a_{4,1} \zeta - a_{4,2} x - a_{4,3} y - a_{4,4} z\\ \end{array} \right] \end{aligned}$$

**Fig. 11:**
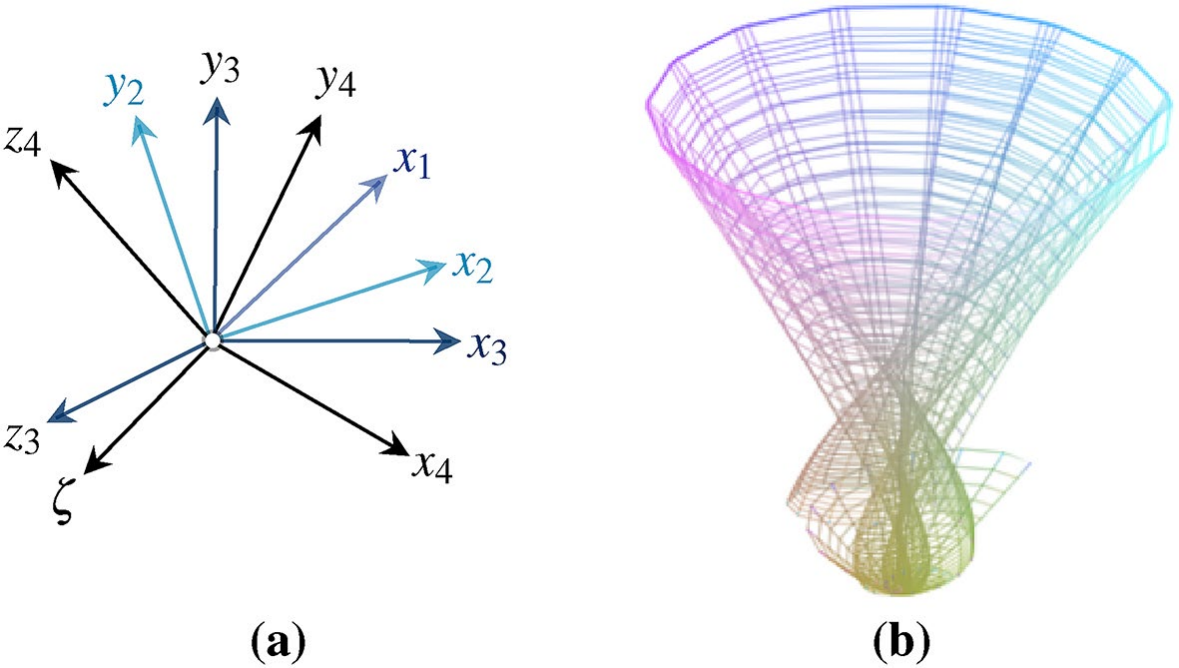
The figure shows the paraboloidal result (**b**) of the cross-dimensional coordinate system shown in (**a**) which uses the spatial directions of $\{ \mathbb {R}^{1}: x \}$ and $\{ \mathbb {R}^{2}: x, y \}$ across the dimensions.

In the manifold of $\mathbb {R}^{3}$, the underlying and superordinate inertial frames of different dimensions pass through the same initial point. We summarize the diagrams of Fig. 10 together in (a) of Fig. 11.

The illustration (b) of Fig. 11 is modeled as a parabolic plane by Eq. 37 to illustrate the metric as three-dimensional orthogonal coordinates. This kind of curvilinear coordinate system indicates the symmetrical behavior around the axis. We form the characteristic polynomial of (b) corresponding to $\{ \lambda ^{4}+\left( ze _{z}- ye _{y}- xe _{x}\right) \lambda ^{3}- xe _{x} ze _{z} \lambda ^{2}- ye _{y} ze _{z} \varsigma e_{\varsigma } \lambda + xe _{x} ye _{y} ze _{z} \varsigma e_{\varsigma } \}$ of Eq. 42.

Our example shows one way of representing the cross-dimensional equation system relating to the x-direction. Overall, there are different commutative paths of cross-dimensional spaces as Table 1 shows.

**Table 1:** The illustration defines the structure of cross-dimensional spaces.

	Refer to x	Refer to y	Refer to z	
(a) $\mathbb {R}^{1}:$	*x*	*y*	*z*	
(b) $\mathbb {R}^{2}:$	*xy* or *xz*	*yx* or *yz*	*zy* or *zx*	
(c) $\mathbb {R}^{3}:$	*xyz*	*xyz*	*xyz*	
(d) $\mathbb {R}^{4}:$	$\zeta xyz$	$\zeta xyz$	$\zeta xyz$	

The 1st dimension specifies the reference direction. There are two reference variants in the dimension of $\mathbb {R}^{2}$. The 3rd and 4th dimensions include all possible spatial directions.

**Fig. 12:**
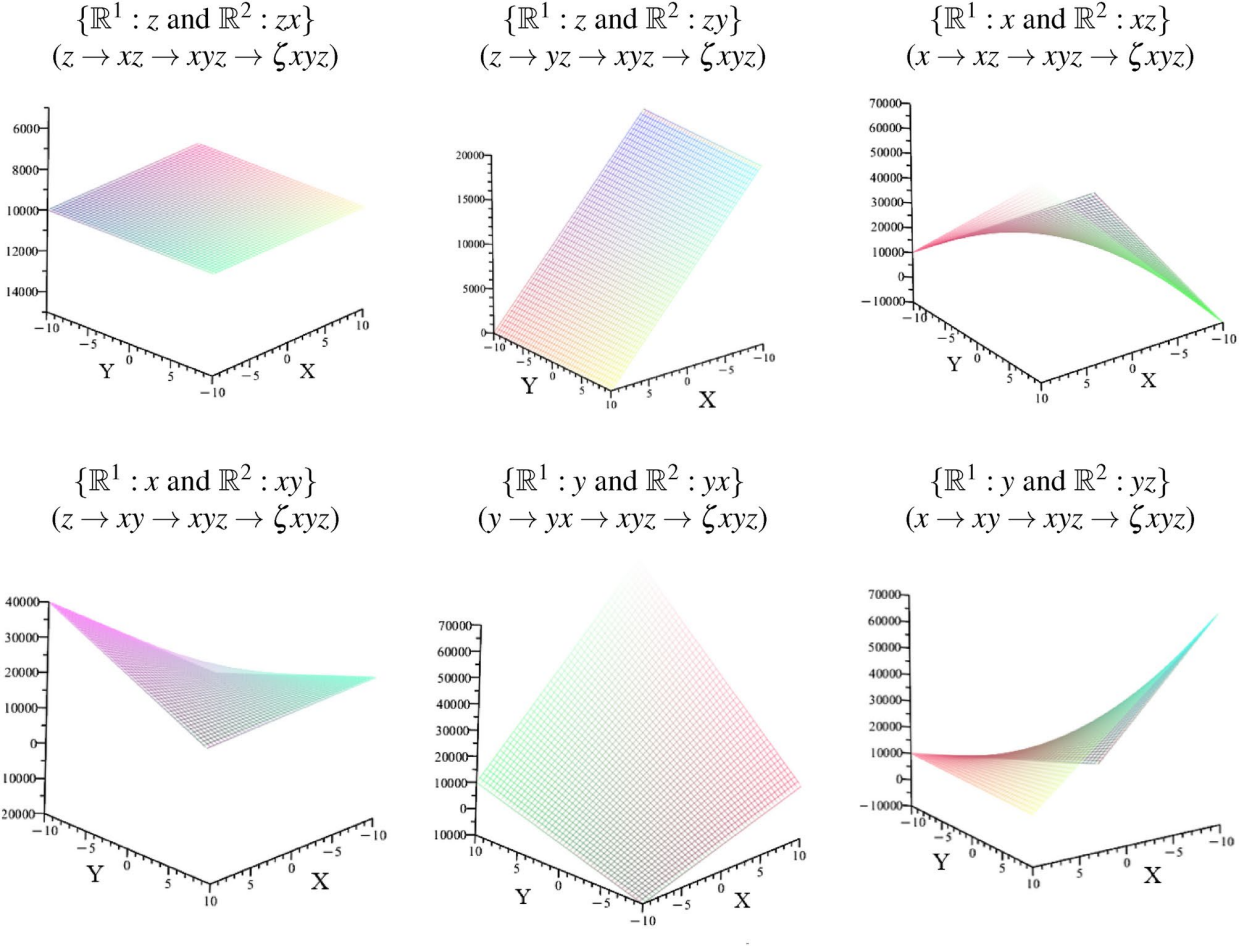
The illustration shows various planes in the manifold of $\mathbb {R}^{3}$ which refer to the cross-dimensional structures of Table 1. The planes reveal a significant shape that depends on its reference direction.

The commutative arrays of cross-dimensional spaces can be expressed as a characteristic polynomial that corresponds to specified planes, as shown in Fig. 12.

The illustrations of Fig. 12 are modeled by the polynomials of Eq. 38 - Eq. 43.

38 $$\begin{aligned} & \{ \mathbb {R}^{1}:z~and~ \mathbb {R}^{2}: zx \}:~ \lambda ^{4}+\left( ze _{z}- xe _{x}- ye _{y}\right) \lambda ^{3}+\left( ye _{y} xe _{x}- ze _{z} \varsigma e_{\varsigma }\right) \lambda ^{2}\nonumber \\ & \quad +\left( xe _{x} ze _{z} \varsigma e_{\varsigma }+ ye _{y} ze _{z} \varsigma e_{\varsigma }\right) \lambda - xe _{x} ye _{y} ze _{z} \varsigma e_{\varsigma } \end{aligned}$$

39 $$\begin{aligned} & \{ \mathbb {R}^{1}:z~ and ~\mathbb {R}^{2}: zy \}:~\lambda ^{4}+\left( - ye _{y}+ ze _{z}\right) \lambda ^{3}+\left( - ye _{y} xe _{x}+ xe _{x} ze _{z}- ze _{z} \varsigma e_{\varsigma }\right) \lambda ^{2}\nonumber \\ & \quad +\lambda ye _{y} ze _{z} \varsigma e_{\varsigma }+ xe _{x} ye _{y} ze _{z} \varsigma e_{\varsigma } \end{aligned}$$

40 $$\begin{aligned} & \{ \mathbb {R}^{1}:x~ and ~\mathbb {R}^{2}: xz \}:~ \lambda ^{4}+\left( ze _{z}- xe _{x}- ye _{y}\right) \lambda ^{3}+ ye _{y} xe _{x} \lambda ^{2}-\varsigma e_{\varsigma } xe _{x} ze _{z} \lambda + xe _{x} ye _{y} ze _{z} \varsigma e_{\varsigma } \end{aligned}$$

41 $$\begin{aligned} & \{ \mathbb {R}^{1}:x~ and ~\mathbb {R}^{2}: xy \}:~ \lambda ^{4}+\left( ze _{z}- ye _{y}- xe _{x}\right) \lambda ^{3}- xe _{x} ze _{z} \lambda ^{2}- xe _{x} ye _{y} ze _{z} \varsigma e_{\varsigma } \end{aligned}$$

42 $$\begin{aligned} & \{ \mathbb {R}^{1}:y~ and ~\mathbb {R}^{2}: yx \}:~ \lambda ^{4}+\left( ze _{z}- ye _{y}- xe _{x}\right) \lambda ^{3}- xe _{x} ze _{z} \lambda ^{2}- ye _{y} ze _{z} \varsigma e_{\varsigma } \lambda + xe _{x} ye _{y} ze _{z} \varsigma e_{\varsigma } \end{aligned}$$

43 $$\begin{aligned} & \{ \mathbb {R}^{1}:y~ and ~\mathbb {R}^{2}: yz \}:~ \lambda ^{4}+\left( ze _{z}- ye _{y}\right) \lambda ^{3}+\left( - ye _{y} xe _{x}+ xe _{x} ze _{z}\right) \lambda ^{2}\nonumber \\ & \quad - ye _{y} ze _{z} \varsigma e_{\varsigma } \lambda - xe _{x} ye _{y} ze _{z} \varsigma e_{\varsigma } \end{aligned}$$

In Fig. 12 , we can see that the cross-dimensional metric of the respective direction differs significantly in the surrounding geometries. Depending on the spatial orientation of this metric, the geometric planes exhibit a different curvature behavior.

**Fig. 13:**
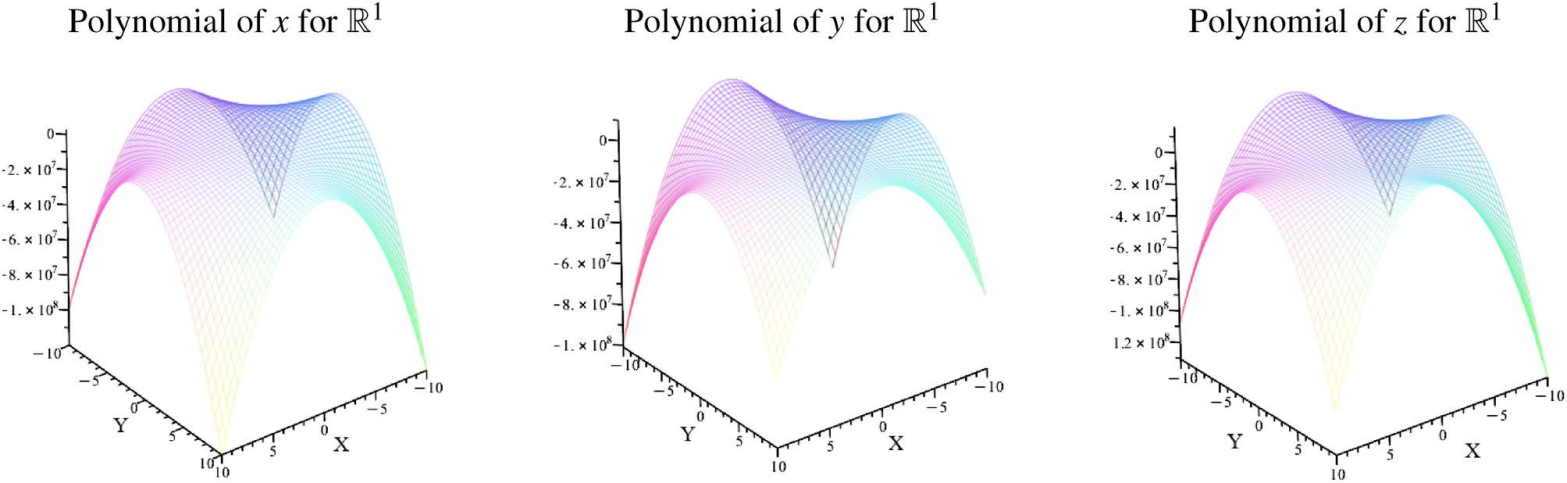
The illustration describes the resulting planes that are summarized by all possible spatial directions. For the x direction, xz and xz om $\mathbb {R}^{1}$ were combined. For the y direction, yx and yz om $\mathbb {R}^{1}$ were combined. For the z direction, xz and yz om $\mathbb {R}^{1}$ were combined.

To verify whether the cross-dimensional equations from the respective reference directions are identical to each other, we form the resulting equations from the possible spatial directions for x, y, and z. The modeled result of the verification is shown in Fig. 13.

The illustrations of Fig. 13 are modeled by the polynomials of Eq. 45 - Eq. 46. The figure indicates the resulting planes of cross-dimensional metric in *x*, *y* and *z* direction.

$$\begin{aligned} & \text {Characteristic~polynomial~of~x-referred~direction~in}~\mathbb {R}^{1}:\\ & \quad (x \rightarrow xy \rightarrow xyz \rightarrow \zeta xyz )~ \& ~(x \rightarrow xz \rightarrow xyz \rightarrow \zeta xyz ) \\ & \lambda ^{4}+\left( - xe _{x}^{2}- ye _{y} xe _{x}+ xe _{x} ze _{z}- ye _{y}^{2}+2 ye _{y} ze _{z}- ze _{z}^{2}\right) \lambda ^{3}\\ & \quad +\left( - xe _{x}^{2} ye _{y} ze _{z}- xe _{x}^{2} ze _{z} \varsigma e_{\varsigma }- xe _{x} ye _{y} ze _{z} \varsigma e_{\varsigma }+ ze _{z}^{2} \varsigma e_{\varsigma } xe _{x}\right) \lambda ^{2}+ \end{aligned}$$

44 $$\begin{aligned} \left( - xe _{x}^{2} ye _{y}^{2} ze _{z} \varsigma e_{\varsigma }- xe _{x}^{2} ye _{y} ze _{z}^{2} \varsigma e_{\varsigma }\right) \lambda - xe _{x}^{2} ye _{y}^{2} ze _{z}^{2} \varsigma e_{\varsigma }^{2} \end{aligned}$$

$$\begin{aligned} & \text {Characteristic~polynomial~of~y-referred~direction~in}~\mathbb {R}^{1}:\\ & \quad (y \rightarrow yx \rightarrow xyz \rightarrow \zeta xyz )~ \& ~(y \rightarrow yz \rightarrow xyz \rightarrow \zeta xyz ) \\ & ~\lambda ^{4}+\left( -2 xe _{x} ye _{y}+ xe _{x} ze _{z}- ye _{y}^{2}+2 ye _{y} ze _{z}- ze _{z}^{2}\right) \lambda ^{3}\\ & \quad +\left( xe _{x}^{2} ye _{y} ze _{z}- xe _{x}^{2} ze _{z}^{2}- xe _{x} ye _{y} ze _{z} \varsigma e_{\varsigma }-2 ye _{y}^{2} ze _{z} \varsigma e_{\varsigma }+2 ye _{y} ze _{z}^{2} \varsigma e_{\varsigma }\right) \lambda ^{2}+ \end{aligned}$$

45 $$\begin{aligned} \left( - xe _{x}^{2} ye _{y}^{2} ze _{z} \varsigma e_{\varsigma }+2 xe _{x}^{2} ye _{y} ze _{z}^{2} \varsigma e_{\varsigma }- ye _{y}^{2} ze _{z}^{2} \varsigma e_{\varsigma }^{2}\right) \lambda - xe _{x}^{2} ye _{y}^{2} ze _{z}^{2} \varsigma e_{\varsigma }^{2} \end{aligned}$$

$$\begin{aligned} & \text {Characteristic~polynomial~of~z-referred~direction~in}~\mathbb {R}^{1}:\\ & \quad (z \rightarrow xz \rightarrow xyz \rightarrow \zeta xyz )~ \& ~(z \rightarrow yz \rightarrow xyz \rightarrow \zeta xyz ) \\ & ~\lambda ^{4}+\left( - xe _{x}^{2}- xe _{x} ye _{y}- xe _{x} ze _{z}- ye _{y}^{2}-2 ye _{y} ze _{z}- ze _{z}^{2}\right) \lambda ^{3}+\\ & \quad \left( xe _{x}^{2} ye _{y} ze _{z}+ xe _{x}^{2} ze _{z} \varsigma e_{\varsigma }+ xe _{x} ye _{y} ze _{z} \varsigma e_{\varsigma }+ xe _{x} ze _{z}^{2} \varsigma e_{\varsigma }\right) \lambda ^{2}+ \end{aligned}$$

46 $$\begin{aligned} \left( xe _{x}^{2} ye _{y}^{2} ze _{z} \varsigma e_{\varsigma }- xe _{x}^{2} ye _{y} ze _{z}^{2} \varsigma e_{\varsigma }\right) \lambda - xe _{x}^{2} ye _{y}^{2} ze _{z}^{2} \varsigma e_{\varsigma }^{2} \end{aligned}$$

Suppose we consider all possible metrics of a spatial direction in one equation, like for example $(x \rightarrow xy \rightarrow xyz \rightarrow \zeta xyz )~ \& ~(x \rightarrow xz \rightarrow xyz \rightarrow \zeta xyz)$. In that case, the spatial directions converge in their functional behavior (as shown in Eq. 45 - Eq. 46 and the illustrations in Fig. 13), but they are not identical. The modeling already shows that the curved edges are not symmetrical with respect to the preferred directions *x*, *y* and *z*. Compared to the geometric planes from Fig. 12, this is also quite understandable, as the metric for the x and y directions shows a curved plane which, however, does not exist in the z direction.

**Fig. 14:**
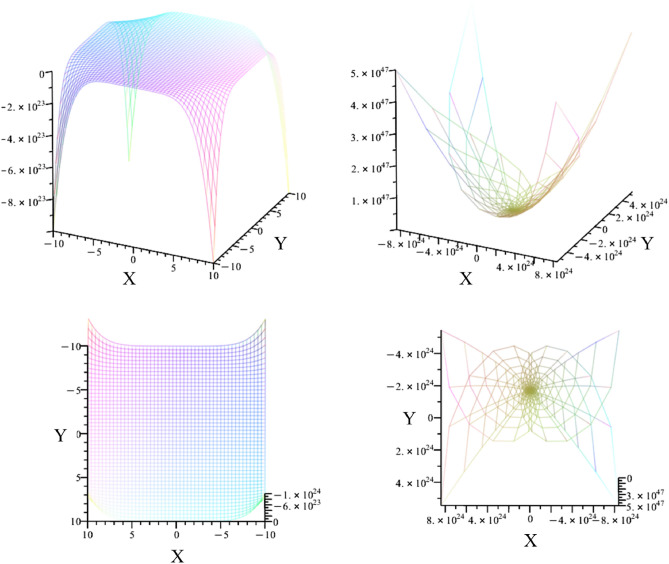
The illustration shows the modeled result of Eq. 47. On the left, the resulting plane is plotted in Cartesian form. On the right, the resulting plane is plotted in parabolic form.

By multiplying all cross-dimensional possibilities, as shown in Eq. 47, we obtain the symmetrical shape in Fig. 14.

$$\begin{aligned} \left[ \begin{array}{c c c c} 0 & 0 & 0 & z e_{z} \\ 0 & x e_{x} & 0 & z e_{z} \\ 0 & x e_{x} & y e_{y} & z e_{z} \\ \zeta e_{z} & - x e_{x} & - y e_{y} & - z e_{z} \end{array} \right] \left[ \begin{array}{c c c c} 0 & 0 & 0 & z e_{z} \\ 0 & 0 & y e_{y} & z e_{z} \\ 0 & x e_{x} & y e_{y} & z e_{z} \\ \zeta e_{z} & - x e_{x} & - y e_{y} & - z e_{z} \end{array} \right] \left[ \begin{array}{c c c c} 0 & x e_{x} & 0 & 0 \\ 0 & x e_{x} & 0 & z e_{z} \\ 0 & x e_{x} & y e_{y} & z e_{z} \\ \zeta e_{z} & - x e_{x} & - y e_{y} & - z e_{z} \end{array} \right] \end{aligned}$$

47 $$\begin{aligned} \left[ \begin{array}{c c c c} 0 & x e_{x} & 0 & 0 \\ 0 & x e_{x} & y e_{y} & 0 \\ 0 & x e_{x} & y e_{y} & z e_{z} \\ \zeta e_{z} & - x e_{x} & - y e_{y} & - z e_{z} \end{array} \right] \left[ \begin{array}{c c c c} 0 & 0 & y e_{y} & 0 \\ 0 & x e_{x} & y e_{y} & 0 \\ 0 & x e_{x} & y e_{y} & z e_{z} \\ \zeta e_{z} & - x e_{x} & - y e_{y} & - z e_{z} \end{array} \right] \left[ \begin{array}{c c c c} 0 & 0 & y e_{y} & 0 \\ 0 & 0 & y e_{y} & z e_{z} \\ 0 & x e_{x} & y e_{y} & z e_{z} \\ \zeta e_{z} & - x e_{x} & - y e_{y} & - z e_{z} \end{array} \right] \end{aligned}$$

The resulting Cartesian geometry in Fig. 14 shows a flat plane that bends symmetrically towards the edges.

Fig. 14 shows the Cartesian and parabolic modeling of the resulting Eq. 47. The Cartesian geometry is characterized by a plane that curves towards the edges. In the parabolic representation, the curvature is inverse. Compared to the planes of Fig. 13, the plane of Fig. 14 has a symmetric structure.

## Results of 4D perception in relativistic image processing

The introduced content of tensor-based space and cross-dimensional metric provides a novel approach to compute 4D motion by sensor information in relativistic image processing. Spatial and temporal correlations are linked with image and sensor information. This offers a new way to calculate depth information and the solvability of a well-known problem from computer vision: Correspondence problem^43,44^. This problem relates to the correct searching and matching of features in stereoscopic images to estimate the correct depth.

The following sections discuss our results of sensor perception and the associated extraction of temporal-related features and depth information. We introduce the technique of image-based superimposition to analyze the relationships between features, perspective, positions, time, and cross-dimensional metric. Image superimposition allows us to relate temporally consistent features geometrically and to increase the detectability of features significantly. In this context, we also present the usability of sensor maps in which sensor data is processed image-related as color information.

### Feature-based superimposition

An important part of computer vision is the recognition, classification, and segmentation of features in image-related scenes^44^. Conventional detection algorithms, such as Features Accelerated Segment Test (FAST), Binary Robust Invariant Scalable Keypoints (BRISK), Oriented and Rotated BRIEF (ORB), and Scale-Invariant Feature Transform (SHIFT)^45^ identify features from corners and edges in contour-rich images^43^. These features can be compared stereoscopically to extract depth information. The recognizability and assignability of such features are related to the image properties and quality. Insufficient contrasts and textures limit the recognizability and assignability of these features^46–48^. If there is also a lack of stereoscopic conditions^46^, no depth information can be extracted.

To increase the density of features, we introduce the image superimposition. Some preliminary work indicates that the superimposition enables the decomposition of the image, and the correction of reflections and illumination^49–51^. The superimposition of scene-dependent images offers perspective changes over time. This also means that the contours become more prominent in the state of motion, which affects the intensity of the features. The superimposition $N_{i}$ can be derived from the local maxima and minima of two images $I_{[i-1]},I_{[i]},$ as shown in Eq. 48.

48 $$\begin{aligned} N_{i} = | max(I_{[i-1]} , I_{[i]}) - min(I_{[i-1]} , I_{[i]}) | + N_{i - 1} \end{aligned}$$

**Fig. 15:**
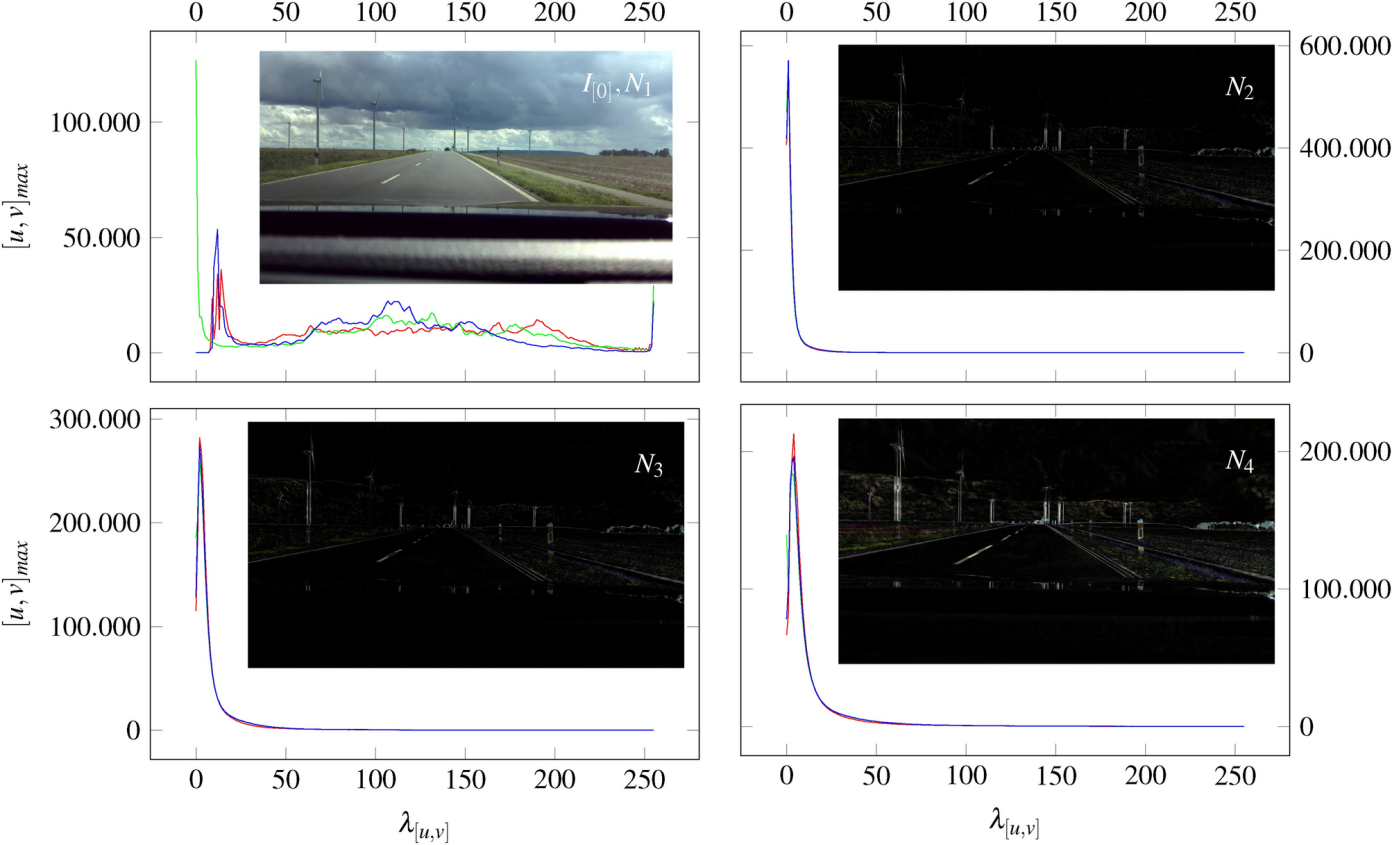
The figure shows the resulting superimpositions $N_{2},N_{3}$ and $N_{4}$ calculated by Eq. 48. $I_{[0]}$ forms the initial image. Further, the change of color values and pixel intensity is illustrated as a function of the image overlays.

By applying Eq. 48, we superimpose 3 images ($N_{1},N_{2},N_{3}$) as Fig. 15 shows. Whereby, $I_{[0]}$ forms the initial images with no superimpositon $N_{0}$.

**Fig. 16:**
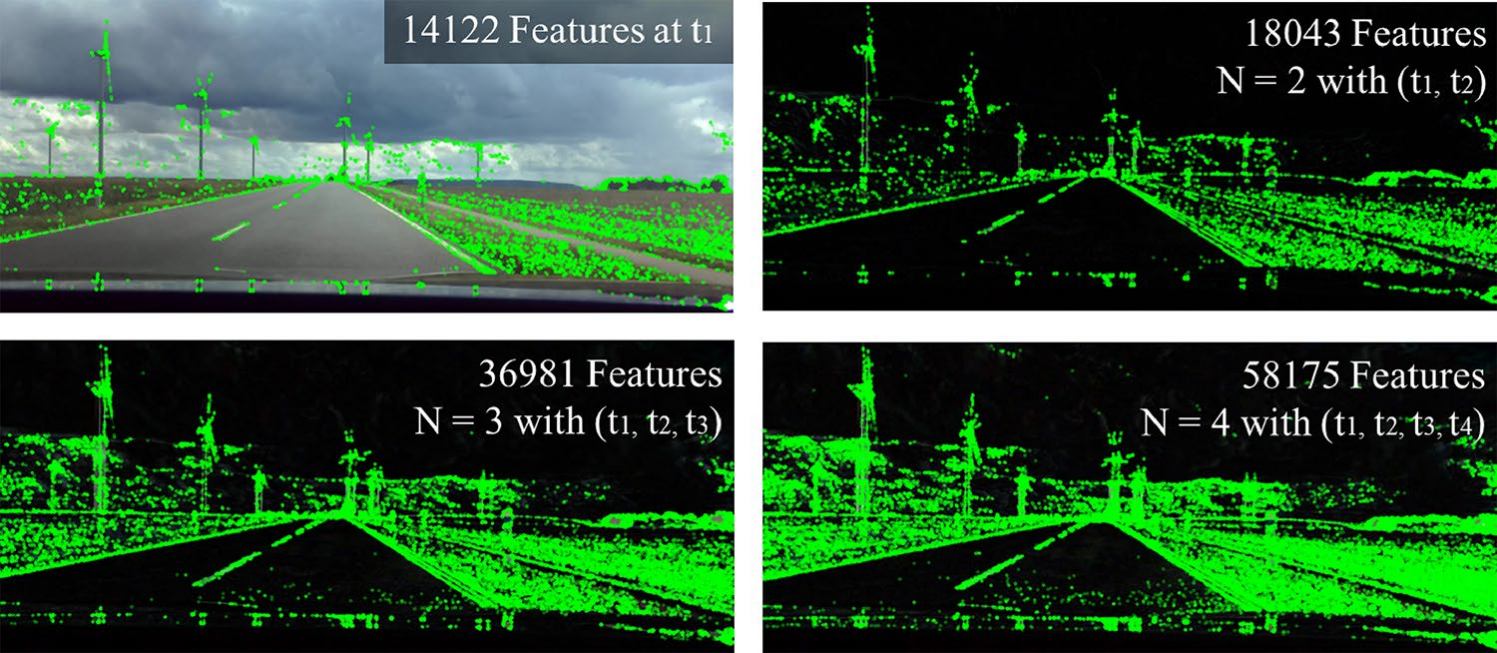
The illustration shows the number of features (green dots) which increases with the number of superimpositions. The features are determined using the FAST algorithm at an image resolution of 1920x1080.

**Fig. 17:**
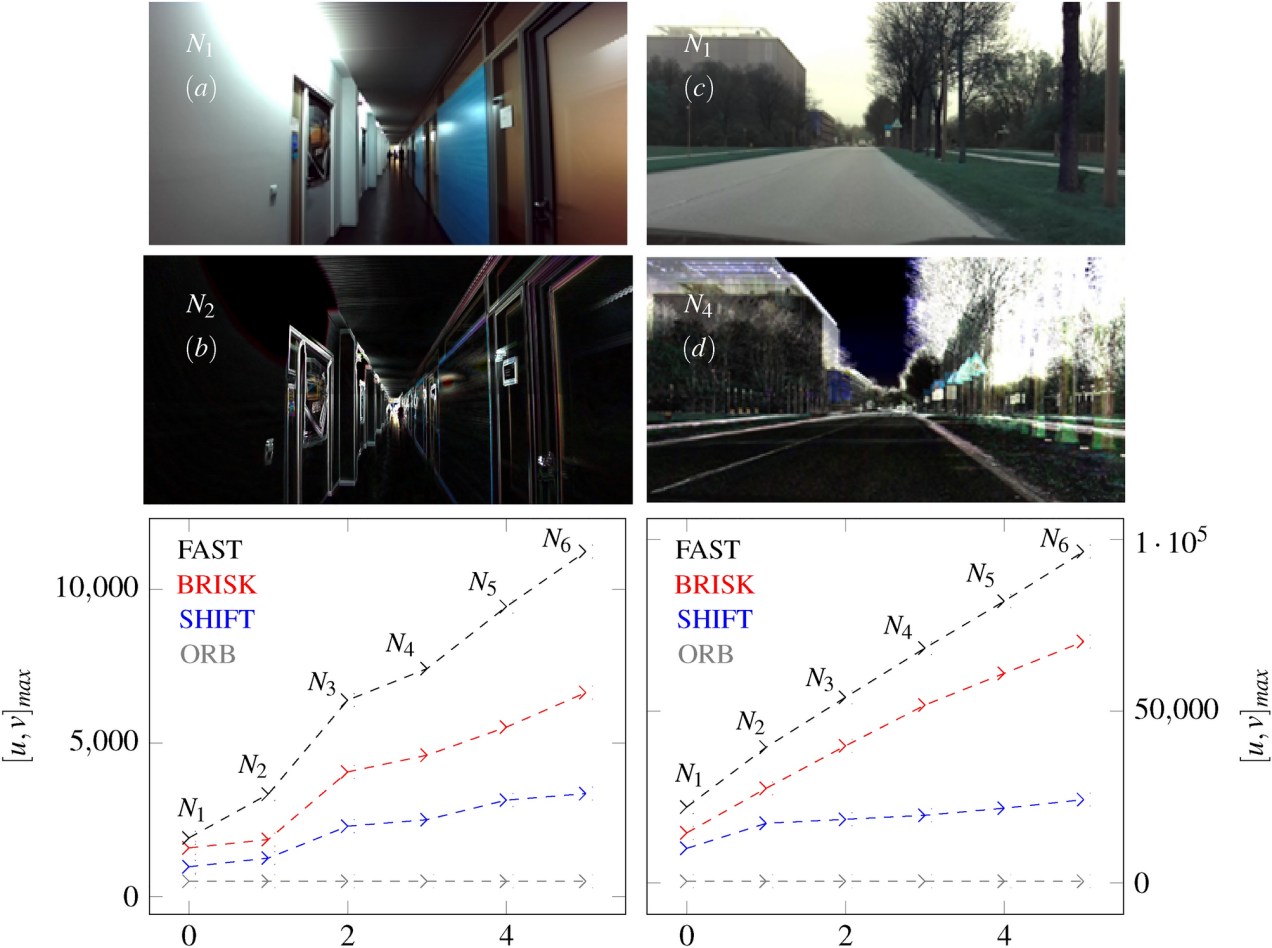
The figure shows the influence of BRISK, SHIFT, FAST and ORB algorithms on the superpositions from indoor (left side) and outdoor (right side) images.

**Fig. 18:**
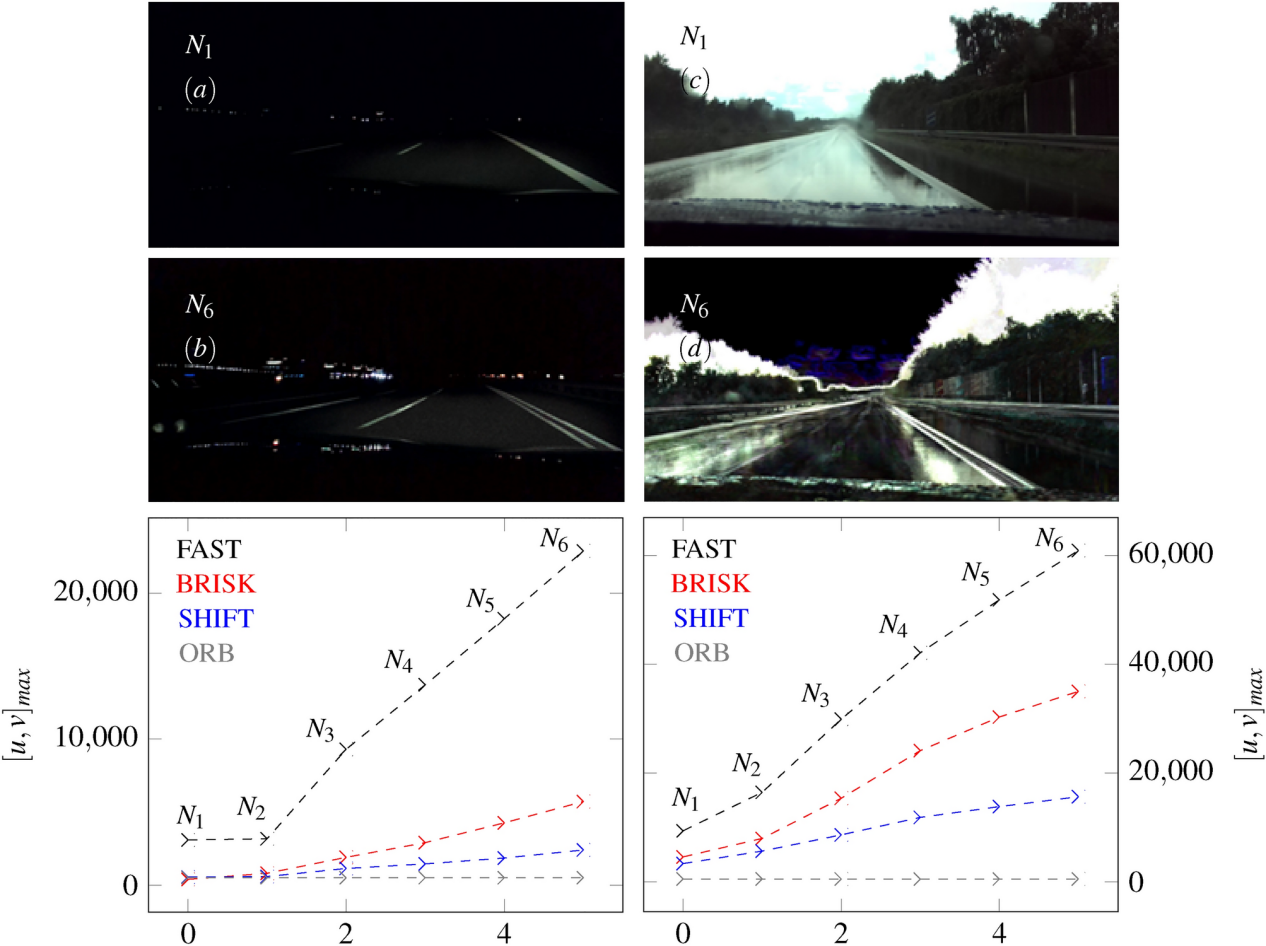
The figure shows the influence of BRISK, SHIFT, FAST and ORB algorithms on the superposition of images at a test drive in the dark ((**a**) on the left side) and under rain conditions ((**c**) on the right side). 6 images of the scenes from (**b**) and (**d**) are superimposed $N_{6}$.

Figure 15 shows the dependencies between the number and intensity of pixels using the principle of image-based superimposition. The curve indicates that the RGB values partially overlap, whereas a significant peak can be characterized. The number of image pixels decreases with the increase in superimposition, measured at the top of this peak. Further, we can see that the superimposition of images reduces the number of color pixels and highlights contours such as corners and edges. This can favor the finding of correct features, which are required in conventional image processing and related to the correspondence problem^52^. To show the influence of image superimposition on feature detection, we consider the images of Fig. 16, Fig. 17 and Fig. 18. The feature identification in Fig. 16 is performed by the FAST algorithm.

Figure 16 shows the benefits of feature identification in superimposed images by FAST-algorithm. We can see that the feature density increases with the increasing number of image overlays. The contours become clearer due to the overlay, which favors recognition density. Further, a comparison of different detection algorithms such as BRISK, SHIFT, FAST and ORB results in an increased feature density, as Fig. 17 and 18 show.

The image on the left (a) in Fig. 17 shows the superimposition of a corridor. Existing light sources and smooth surfaces in the original image cause over-exposures or reflections of individual areas. However, these effects can be reduced by superimposing 2 images $N_{2}$, see (b) in Fig. 17. Furthermore, contours are easily recognizable. The smallest position offsets between the images are sufficient to emphasize the contours. This is also evidenced by the increased curves of feature density in the histogram below the superimposed image. The direct comparison shows that the FAST algorithm detects the most features. The increased detectability can also be seen in other histograms from Fig. 17 and Fig. 18. Whereby the brighter outdoor images contain a higher number of features. A further highlight can be seen in (a) and (b) of Fig. 18. Image overlays can enhance the dark areas of individual images. In (b) the road is better illuminated with an overlay of 6 images ($N_{6}$). The distance to the oncoming traffic on the left side is also recognizable. The improved brightness values can emphasize the contours. The moved images differ in their position and related perspective, which means that the image overlay in (b) has several strips instead of just one strip (a). This effect can also be seen in (d).

Our results show that the superposition of images improves the detectability of features. Images with poor brightness values or a degree of reflection can also be improved with this methodology. The pixel position of the features in the overlay can be matched with the identified features of the original images to increase the accuracy of finding and matching features. However, a possible problem arises when the contours are shifted in perspective and the number of overlays is increased. In this case, multiple contours of one type can exist. We assume that this problem can be solved by referencing and decomposing the images to each other after the superimposed detection of features.

### Correspondence problem: temporal-based search of corresponding features by cross-dimensional metric

Modern computer vision is able to calculate depth information by matching features in stereoscopic images. Thereby, the rich textures and contrasts of camera images allows us to percieve numerous details of the environment. The detection algorithms identify points, areas, or patterns within these images and derive features from them^43,47,48^. The matching features can be extracted as a depth map in form of a reciprocal disparity map. This allows us to render point clouds by the trigonometric relationships of the camera and the disparity map.

The matching of identical features proves to be a challenging part of correspondence problem if many pixels have similar color and brightness values, shadow effects or reflections occur or objects are obscured^43,52^. Distortions or parametric deviations of the camera make the assignment of features more difficult which also accompanies with the dynamic changes in position of the camera. Further, the stereoscopic conditions like the distances between the camera images must be considered.

The results of relativistic image processing offer a novel approach to finding and correctly assigning correpsondence features. The distances between associated features can be determined from the geometric relationships of a superimposed image. These features are spatially and temporally related to other sensory information. The cross-dimensional metric acts here as a kind of mask to create the necessary stereoscopic reference for calculating the depth information.

**Fig. 19:**
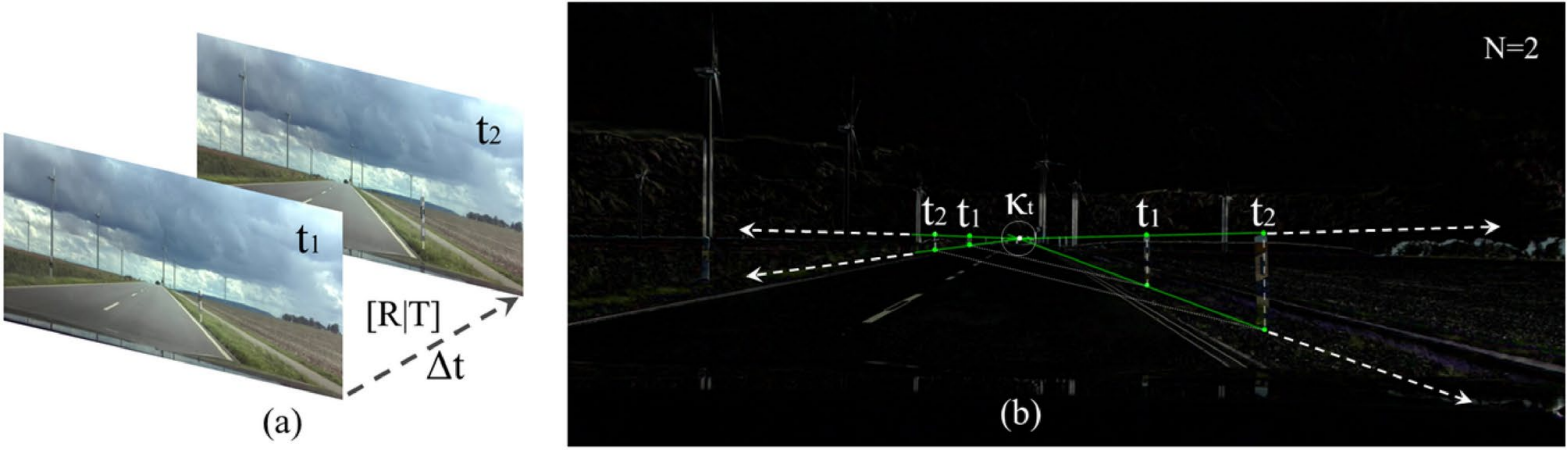
The illustration describes the superimposition of images. The camera moves at a speed of approximately 70 *km*/*h*. In (**a**), two images from different times of a scene are shown. The superimposition in (**b**) shows the positional change as a colored contour which occurs by motion. Regions with minimal changes remain black.

To illustrate what relationships exist between the corresponding features, time, and geometry, we superimpose two images of a scene from two different times ($N=2$) in Fig 19.

Figure 19 describes the visual relationship of positional and perspective changes over time. Our vehicle including the camera moves forward with approximately 70 *km*/*h*. The delineators on the right and left side are at rest.

**Fig. 20:**
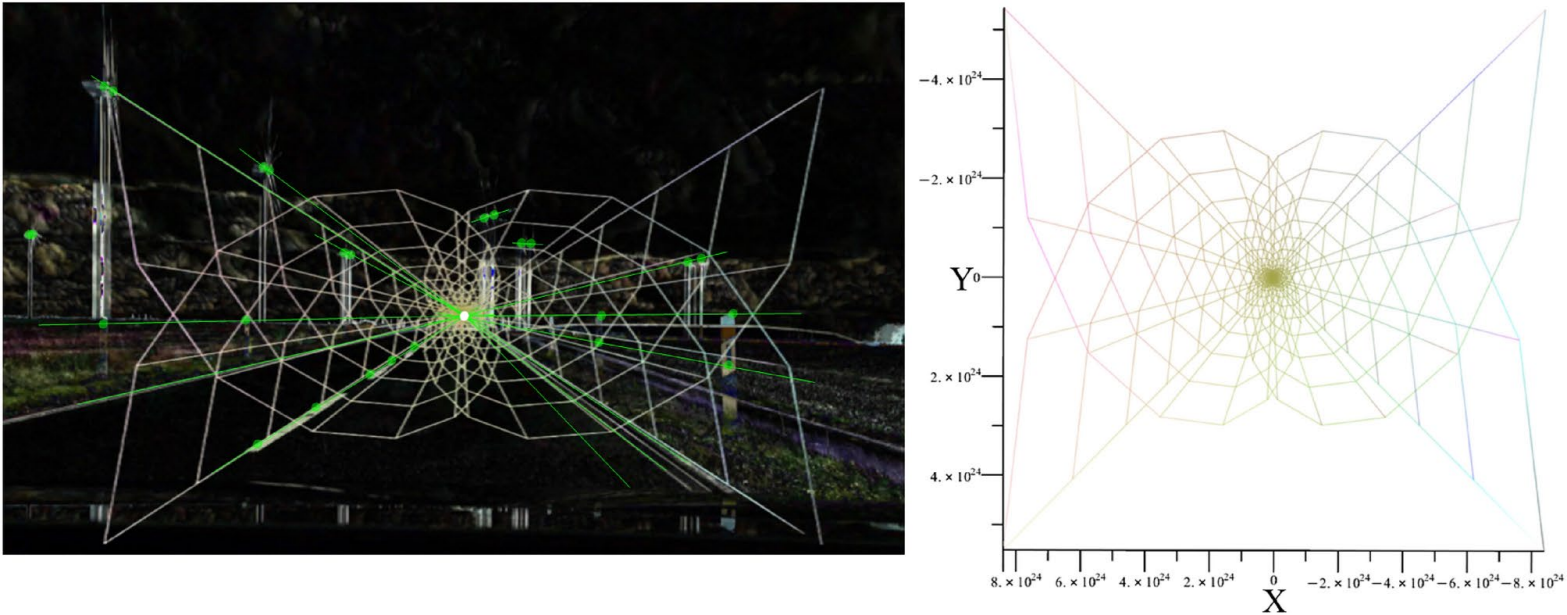
The parabolic geometry of Fig. 14, which corresponds to the cross-dimensional metric, is used as a mask for the superimposition to perspective relationships of corresponding features. The green dots show features of different times which are subjected to the geometric and perspective structures.

The vanishing point $\kappa _{t}$ in Fig. 19 can be determined by superimposing the images and geometrically assigning features. Extracted features from different times, which can be identified in the superimposed image, are geometrically related to each other. We can see the new position of surrounding objects in the image, such as the delineators at the roadside. Due to the forward motion, the static delineators shift geometrically from the image center $t_{1}$ to the edge $t_{2}$. If we connect the features from the same time, we can see that the lines are aligned parallel to each other. Further, if we use the parabolic geometry from Fig. 14 as a mask, we can see that the time-extracted features are located on the geometric lines (shown in Fig. 20).

**Fig. 21:**
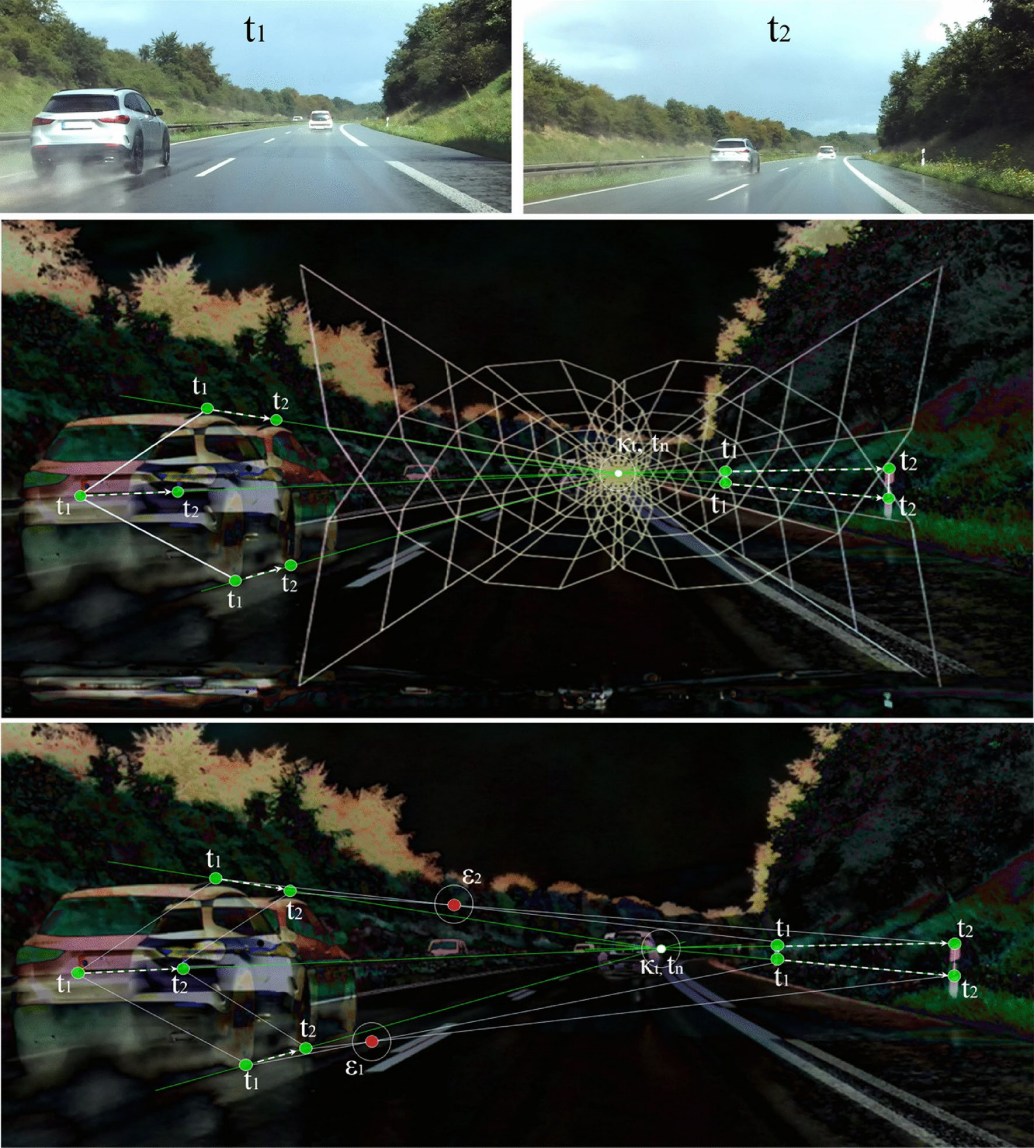
The illustration describes the perspective relationships of corresponding features in the moved state. The camera moves at a speed of approximately 100 *km*/*h*.

The corresponding features in Fig. 19 and Fig. 20 are marked as green dots. The different lines, reconstructed from these corresponding features, cross at the perspective vanishing point. If we superimpose the parabolic mask (Eq. 47 and Fig. 20), we can see that the geometric structures correspond fundamentally with the feature-corresponding lines. This indicates that Eq. 47 can benefit the estimation of depth in image processing to render point clouds as it indicates the perspective progression of dynamic images. The feature distance along the green line within the mask indicates whether an object is near or far. If the corresponding features are close to each other, the object is further away from the camera. However, if the corresponding features are further apart, the object is closer to the camera. It is similar to a baseline^14,43^ which is often known in the static case of stereoscopic images but unknown in the dynamic case of monocular images. As contours appear during motion adjustments, sensor-based position data can be used as a reference to dimensionalize the point clouds metrically. Next to Fig. 20 where the surrounding objects are in a static state, we illustrate the influence of object motion in Fig. 21.

The centered superimposition in Fig. 21 is determined from the upper reference images at $t_{1}$ and $t_{2}$. A vehicle is overtaking on the left. On the right is a static delineator. Our vehicle drives at approximately 100 *km*/*h*. We can see that the distances of corresponding features between the moving vehicle and the stationary delineators are different. One reason for this is the dynamic of the vehicle related to us (camera position).

While the passing vehicle is moving away from us, the delineators are converging. This can be determined by the distance between the features $t_{1}$ and $t_{2}$. On the overtaking vehicle, the distance falls in comparison to the delineators on the right-hand side. A further aspect relates to the connected lines from the features of a corresponding time. In Fig. 19, we can see that the dashed lines of the corresponding times are parallel to each other when the surrounding objects are not moving. In Fig. 21, however, this changes as soon as one of the object is in motion. The lines $t_{1}$ and $t_{2}$ cross at the red marked point. This point, which we denote as the temporal intersection point, is calculated from 2 connected features of a corresponding time.

It is unclear how far the temporal intersection can be transferred to recorded position data. However, it depends on the perspective, velocity, and position in relation to our camera. The presence of the temporal intersection can be used to determine which objects are in motion. If all objects are at rest, the lines are parallel to each other. If one of the objects is moving forwards, the lines intersect. If one of the objects moves backwards, the lines diverge. Despite the existing dynamics, the distances of corresponding features can be estimated correctly. The metric mask is also consistent with the corresponding features. The determination of temporal intersections could be a new way to investigate dynamic effects, such as optical flow^43,53^, to reduce image distortion.

### Sensor maps in relativistic image processing

The processing of sensor information in images is a visual part of relativistic image processing and the associated sensor perception. In this way, sensor and image data can be related to each other and expressed as a sensor map. The characteristic of a sensor map allows us to identify respective changes in motion as color changes in the map. In turn, the sensor-related color composition of the map can appear as a significant rgb peak of the associated histgoram.

**Fig. 22:**
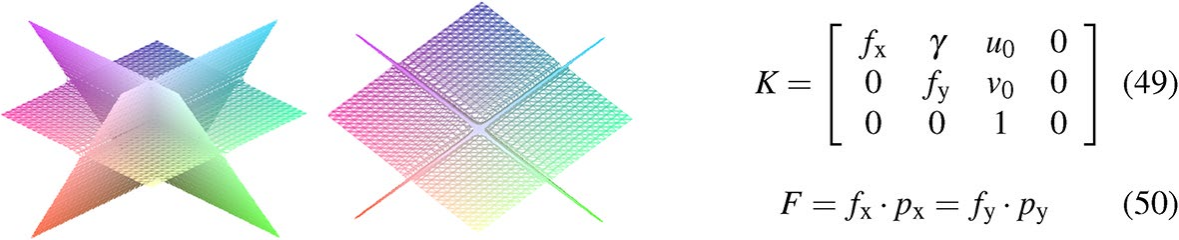
The illustration shows the camera intrinsic planes based on Eq. 49.

We combine images that change their position synchronously with measured sensor information such as accelerations, magnetic fields, or angular velocities. To interpret the composition of sensor maps, we consider the camera-intrinsic projection matrix that transfers images from $\mathbb {R}^{2} \rightarrow \mathbb {R}^{3}$, shown in Fig. 22.

The intrinsic parameters can be determined by the pixel-based focal length $f_\textrm{x},f_\textrm{y}$, optical center $u_\textrm{0},v_\textrm{0}$ and skew coefficient $\gamma$ of referenced image^54^. $f_\textrm{x},f_\textrm{y}$ are the ratio between the world unit defined focal length *F* and the pixel sizes $p_\textrm{x},p_\textrm{y}$ (Eq. 50). We can see, that the camera intrinsic matrix consists of 3 planes in space by polynomial characteristic ($K=\lambda ^{3}-\left( f_{x}+f_{y}+1\right) \lambda ^{2}-\left( -f_{x} f_{y}-f_{x}-f_{y}\right) \lambda -f_{x} f_{y}$ where $\gamma = 1$), shown in Fig. 22. The intrinsic camera matrix is a part of the projection matrix *M*, which contains the extrinsic component [*R*|*T*] in terms of positional shift.

51 $$\begin{aligned} M = K [R|T] = K \cdot \chi ^{\mu } = K \cdot (\zeta \cdot \vec {e}_\mathrm {\zeta } + x \cdot \vec {e}_\textrm{x} + y \cdot \vec {e}_\textrm{y} + z \cdot \vec {e}_\textrm{z}) = K \cdot ({R}^{1,4}_{6}|{T}^{1,4}_{4}) e_\textrm{T} = K \cdot (R_{1,3}^{2} R_{ \zeta }| T_{1,3}(1+\tau _{\Gamma }^{2}) ) \delta _{*}^{\nu } \in \mathbb {R}^{1,4} \end{aligned}$$

Considering temporal aspects, the position-related change can be transferred to the projection matrix using sensor data.

52 $$\begin{aligned} & M_{\omega } = K \cdot \chi ^{\mu }_{\omega } = K \cdot (\delta _{\zeta *}^{\nu } \omega _{\zeta } + \delta _{x*}^{\nu } \omega _{x} + \delta _{y*}^{\nu } \omega _{y} + \delta _{z*}^{\nu } \omega _{z} ) \hspace{0.5cm} \{ \langle V \in \mathbb {R}^{3} \rangle :\omega ^{\nu } \rightharpoonup \langle V^{*} \in \mathbb {R}^{4} \rangle :\omega _{*} \end{aligned}$$

53 $$\begin{aligned} & M_{a} = K \cdot \chi ^{\mu }_{a} = K \cdot (\delta _{\zeta *}^{\nu } a_{\zeta } + \delta _{x*}^{\nu } a_{x} + \delta _{y*}^{\nu } a_{y} + \delta _{z*}^{\nu } a_{z} ) \hspace{0.6cm} \{ \langle V \in \mathbb {R}^{3} \rangle : a^{\nu } \rightharpoonup \langle V^{*} \in \mathbb {R}^{4} \rangle : a_{*} \end{aligned}$$

54 $$\begin{aligned} & M_{B} = K \cdot \chi ^{\mu }_{B} = K \cdot ( \delta _{\zeta *}^{\nu } B_{\zeta } + \delta _{x*}^{\nu } B_{x} + \delta _{y*}^{\nu } B_{y} + \delta _{z*}^{\nu } B_{z} ) \hspace{0.3cm} \{ \langle V \in \mathbb {R}^{3} \rangle : B^{\nu } \rightharpoonup \langle V^{*} \in \mathbb {R}^{4} \rangle : B_{*} \end{aligned}$$

Eq. 51 describes the position of an image in terms of time. The projection matrix of the equation can be extended by various sensors as shown in Eq. 52, Eq. 53 and Eq. 54. To fuse the image and sensor data, it is necessary to assign an intensity to each direction of the sensor. In this context, the data equates to an identifiable value of pixel intensity which can be transferred to an image. The pixel intensity of acquired sensor information can be derived by Eq. 55.

55 $$\begin{aligned} \lambda _{x} = \dfrac{\delta _{max}}{2} \cdot (1 - \dfrac{x_{[n-1]}-x_{[n]}}{x_{[n-1]} + x_{[n]}})~~~~~~~\lambda _{y} = \dfrac{\delta _{max}}{2} \cdot (1 - \dfrac{y_{[n-1]}-y_{[n]}}{y_{[n-1]}+y_{[n]}})~~~~~~~\lambda _{z} = \dfrac{\delta _{max}}{2} \cdot (1 - \dfrac{z_{[n-1]}-z_{[n]}}{z_{[n]}+z_{[n-1]}}) \end{aligned}$$

The sensor-dependent shift of pixel intensity $\lambda _{x}, \lambda _{y}$ and $\lambda _{z}$ define the peak position in the derived histogram of a sensor map. In this context, $\delta$ can be used to define the working range of the sensor map for each respective sensor. This is important because calculated intensity values above 256 do not have their own peak in the histogram. Instead, the deflection is at 0. We calculate the sensor map $\Lambda _{t}$ from the averaged intensity $I_{s}$ and the relativistic vector addition $T = (a + b)/(1+(a\cdot b))$ ^33^.

56 $$\begin{aligned} ~ \Lambda _{t} = \dfrac{I_{s}+\lambda }{ 1+(I_{s} \cdot \lambda ) } \hspace{0.5cm} with \hspace{0.5cm} I_{s} = \dfrac{I_{n} + \lambda }{2} \end{aligned}$$

**Fig. 23:**
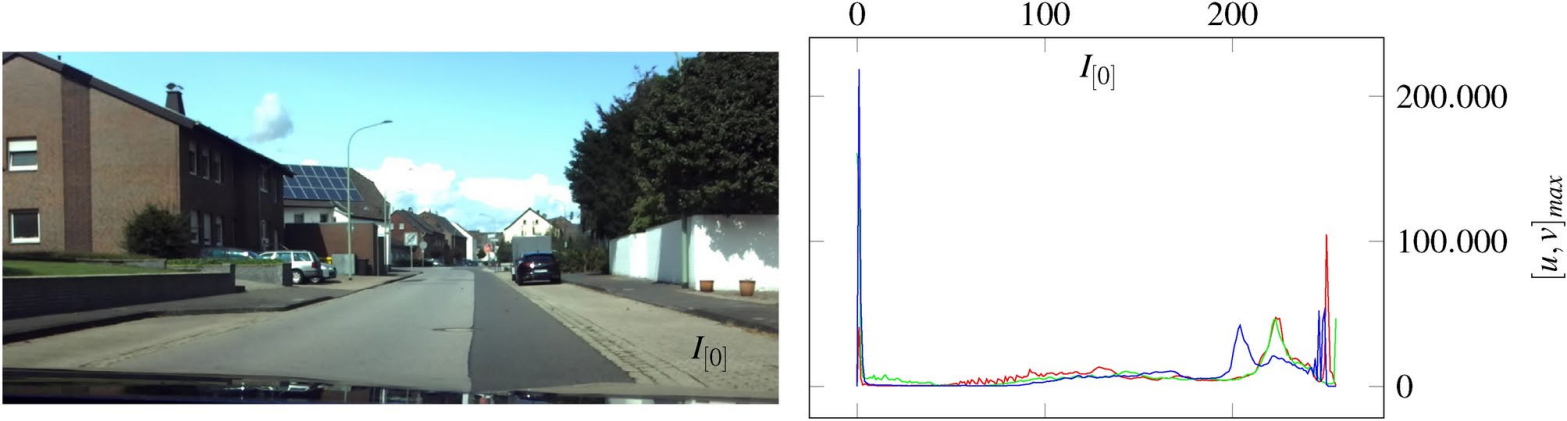
The shown image on the left side is used to calculate the sensor maps from Fig. 25 and Fig. 24. In the figure, the vehicle drives with approximately 30 *km*/*h*. The corresponding histogram can be seen to the right.

**Fig. 24:**
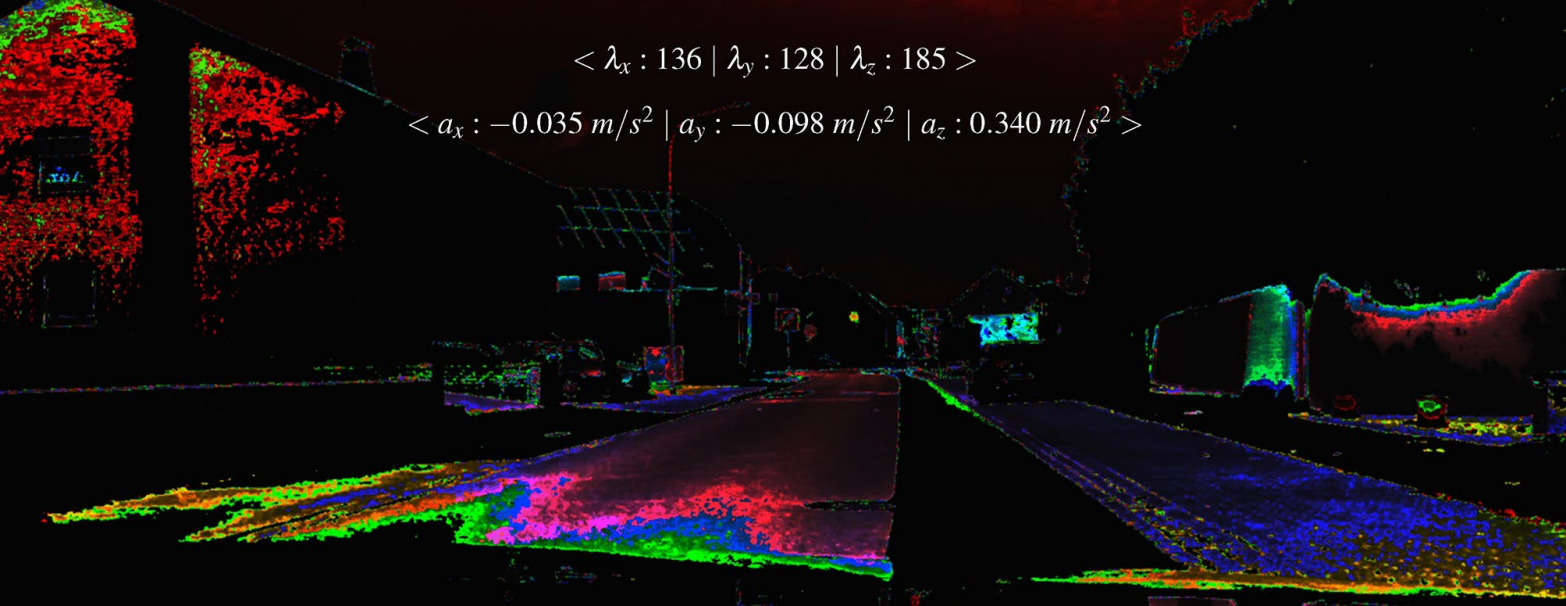
The illustrated sensor map is extracted by the image of Fig. 23 and its associated acceleration data. The different color gradients are derived from the related sensor information, perspective relationships, and properties of the image like brightness, texture, and contrasts.

**Fig. 25:**
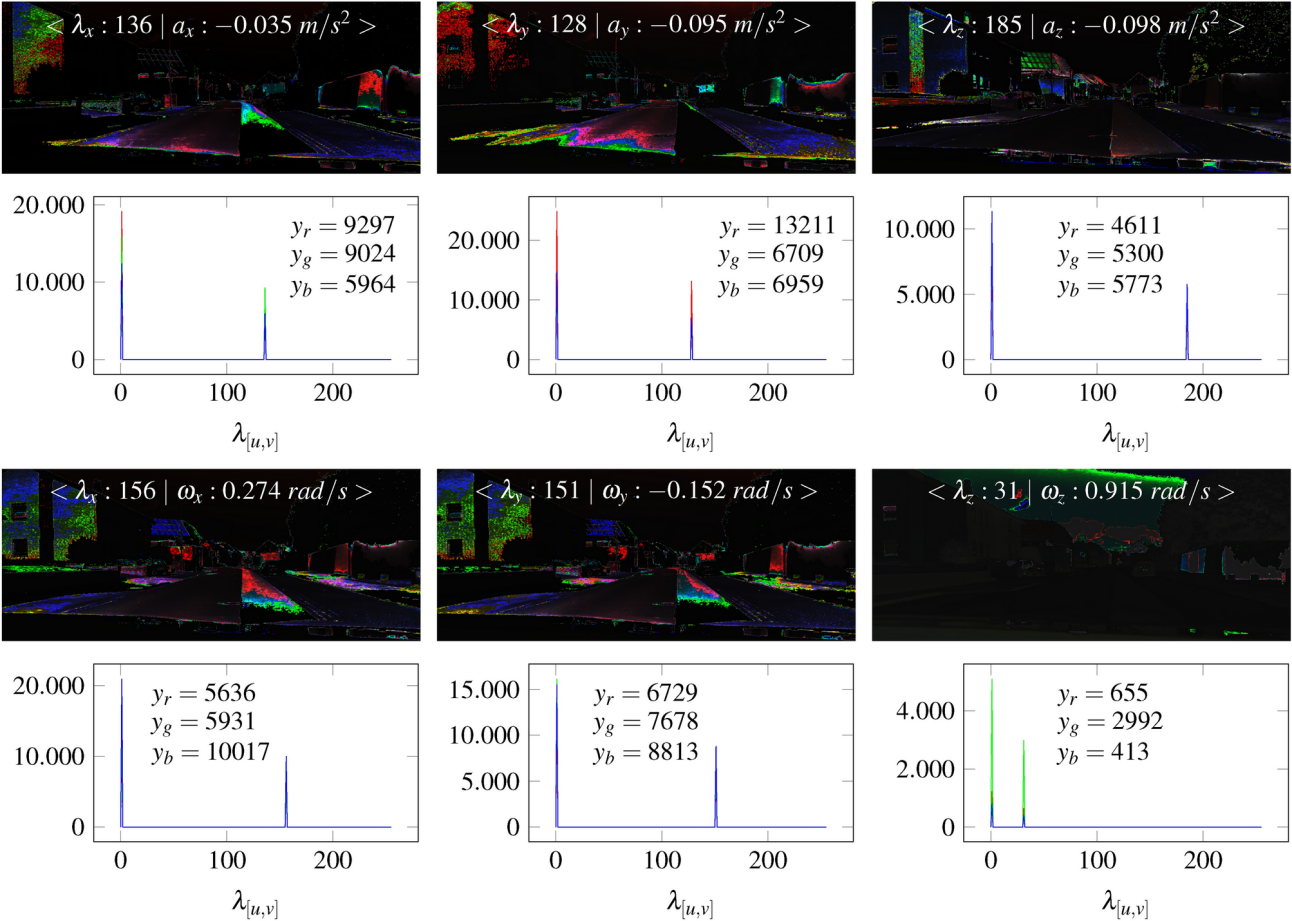
The illustration includes the results of sensor maps and histograms calculated from the acceleration and angular velocity. The data of the upper row comprises the acceleration. In the bottom row, the angular velocity is applied.

The averaged intensity $I_{s}$ is calculated from the current image $I_{n}$ and the sensory intensity $\lambda$. Further, we use the image in Fig. 23 to extract the sensor maps in Fig. 24 and Fig. 25 by Eq. 55 and Eq. 56.

Fig. 23 shows a scenario in which a vehicle including the camera is moving at a velocity of approximately 30  *km*/*h*. The histogram on the right represents the rgb values of the image. Additional data of acceleration and angular velocity is used for the map generation. The following sensor map in Fig. 24 can be extracted from the related image and sensor data.

The individual color gradients of the map in Fig. 24 depends on the respective acceleration and image data. Without adjusting the $\delta$ parameter ($\delta =1$), Eq. 55 results in an intensity value of $<\lambda _{x}:893~|~\lambda _{y}:31~|~\lambda _{z}:1454 >$. $\lambda _{x}$ and $\lambda _{z}$ are consequently outside the intensity spectrum (0 to 256). As a result, the deflections of the respective sensors cannot be assigned and the sensor map remains black. For this reason, we assume a $\delta$ of 256/2. In this case, the intensities of all spatial directions lie within the value range (see Fig. 24). Further, each sensor direction can be characterized by an individual pixel intensity. The pixel intensity itself depends on the sign and amplitude of the sensor data.

Environmental effects such as shadows or reflections (as seen on the wall at right) are filtered in the map. A similar effect can be noticed by superimposing images. The color gradients are derived from the intensity values of the sensor, camera perspective as well as the contrast, light, and texture ratios of the image. We can see that not all areas are marked in color. This is partially dependent on the $\delta$ parameter which defines the working range of the sensor intensity. Furthermore, the slight color gradients or color transitions in z direction are also influenced by $\delta$.

With regard to Fig. 23, the sensor data influences the color gradient and intensity of a sensor map. Fig. 25 shows the dependencies between the color gradients of a sensor map and the specific sensor data comprising its intensity values. All maps are related to the same $\delta$ parameter of 256/2.

The results in Fig. 25 show the relationship between a sensor map and the resulting rgb values in the historgam. All maps have different color gradients which change with the camera and sensor motion. The calculated sensor intensities can be clearly assigned in the histograms. The results show that each intensity generates specific color gradients in the images. Multiple checks also exclude the possibility that the color gradients are randomly generated. The colour gradients in the maps can be recognized as color peaks ($y_{r}:$red, $y_{g}:$ green, $y_{r}:$blue). The peak corresponds to the calculated intensity value of the sensor using Eq. 55. The contours are clear visible by the coloring. The left house illustrates that the color composition varies depending on the direction of motion. For example, although the acceleration in the y direction ($a_{y}=-0.095~m/s^{2}$) and z direction ($a_{z}=-0.098~m/s^{2}$) are close in value, the intensities and rgb distributions are different. In the y direction, the red component dominates with 13211. In the z direction, the blue component dominates with 5773. In our case, the intensity range from 128 to 156 shows stronger color gradients. An intensity value of 38 shows the weakest color gradient.

The direct comparison between the sensor data, intensity value, and rgb values at peak position indicates interesting correlations. Here, the direction of the sensor data and the amount of velocity can already influence the sensor intensity. In this context, it is unclear whether temporal effects in the camera such as the rolling shutter^55^, temporal aliasing^56,57^ influence the sensor map. This also involves verifying the influence of one’s own velocity on the sensor map and the impact of sensor noise.

**Fig. 26:**
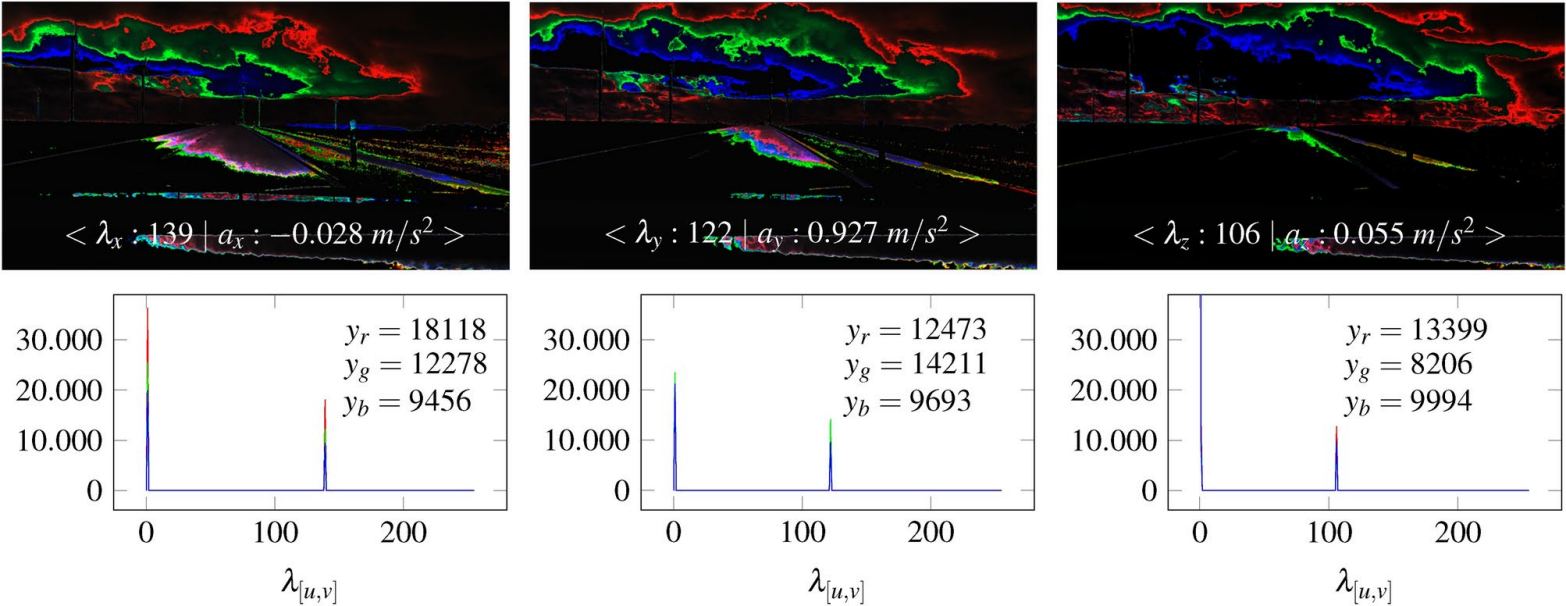
The Illustration shows the different color gradients of a sensor map by a velocity of approximately 70 *km*/*h*. The maps are calculated using the accelerations.

Compared to Fig. 24, Fig. 26 shows that a higher velocity (70 *km*/*h* ) can influence the range of the color gradient in a sensor map. While in Fig. 24 at 30 *km*/*h* the color gradient already starts in the near field, the color gradient in Fig. 26 at 70 *km*/*h* is further away. The acceleration values do not differ significantly from each other.

In addition to the color-based depth progression of the sensor maps in Fig. 26, it can be seen that the accelerations of respective axes differ in the distribution of rgb values. The largest color area along the road appears in the x-direction. The red component dominates with 18118 pixels. Color transitions are more visible along the y-axis. Here, the color green dominates with 14211 pixels. The Z-axis shows the most distant color gradient where the red component dominates the histogram with 13399 pixels. However, the sensor map of the z-direction shows a green color gradient further away along the road.

**Fig. 27:**
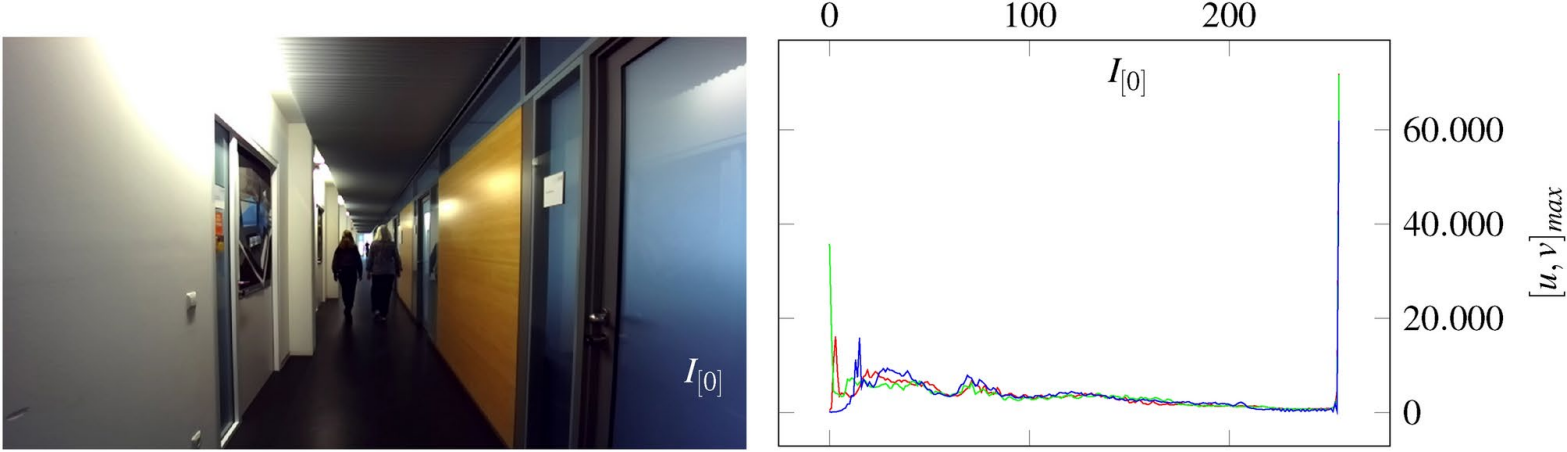
The shown image on the left side is used to calculate the sensor maps from Fig. 28. In the illustration, two people are moving through a corridor. The histrogram on the right refers to the picture.

In addition to sensor maps of different velocities, the influence of object motion shall be illustrated. We consider two people walking along a corridor as shown in Fig. 27. The camera, including the sensors, is at rest.

**Fig. 28:**
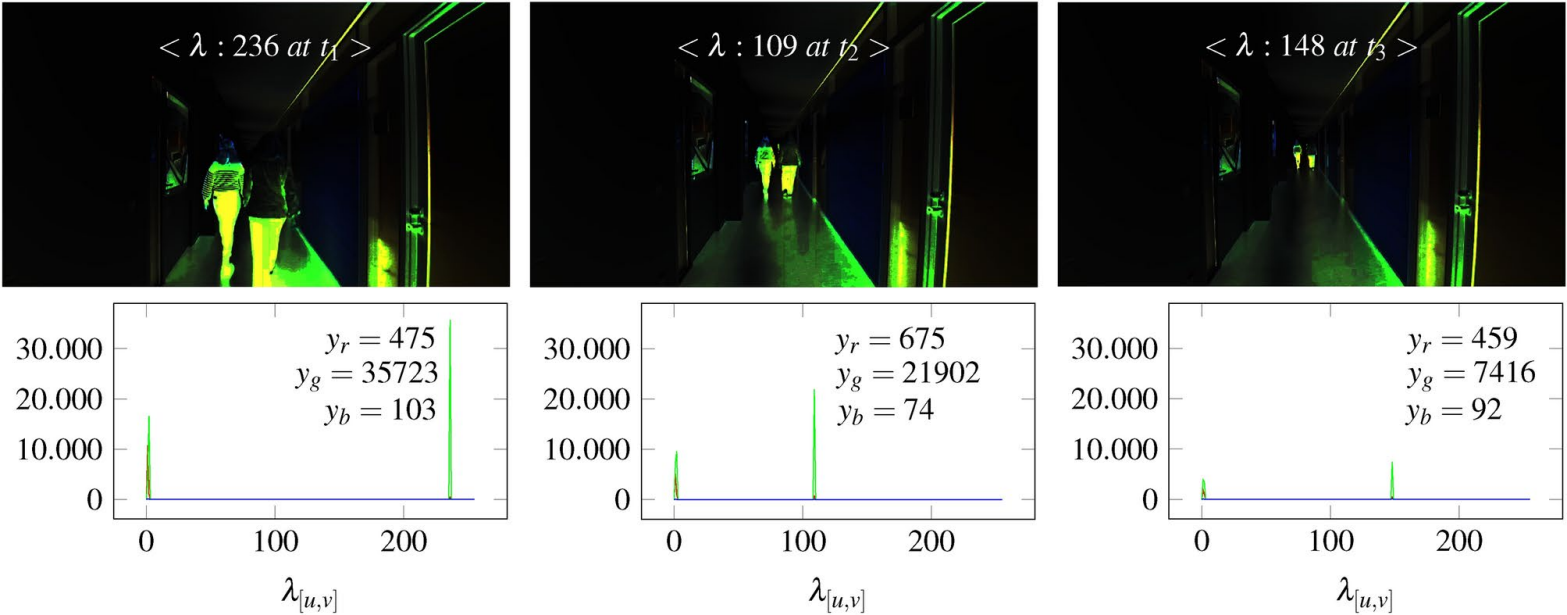
The figure shows the reduction of green color pixels in different sensor maps by moving people. The left image shows a high proportion of green as the people are close. This decreases in the middle image due to the increasing distance. In the right image, the people are far away and the proportion of green is correspondingly low.

Since the accelerations are close to 0 due to the resting state of the camera, we refer to the time. The inclusion of time increases the green ratio in a sensor map, as shown in Fig. 28. This offers the benefit of visualizing object motion. Based on Fig. 27, complementary colors in sensor maps can be highlighted more strongly by adjusting the $\delta$ parameter.

The people are highly visible from both near and far. In this context, a relationship can be defined between the people and the camera. The distance can be determined by measuring the ratio of green in the histogram. We can see that the green component decreases as the distance to the people increases. However, the accuracy can be reduced by reflections or shadows as these are converted to green. This effect can be recognized in the left sensor map of Fig. 27.

The results show that the sensor data can be transferred to a sensor map for spatial perception. The color gradient varies depending on the speed, temporal effects, amplitude, and alignment of the sensor data. The relativistic approaches help to determine sensor intensities. These can be allocated as a unique rgb peak in the histograms. However, further research in the field is needed to find out how the color gradients are related to the properties of sensor and image data. This involves the noise behavior of sensors and properties of the images like resolutions or rectifications. AI approaches could be helpful here to assist with the evaluation of sensor peaks. This in turn would significantly reduce the amount of time required for higher data density.

Further results in which the camera is at rest show that it is possible to perceive objects in color and measure distances as color peaks. In the moving state, the varying color gradients can lead to possible errors when trying to determine object distances. Segmentation or the use of bounding boxes can be helpful here to measure the intensity peaks within the bounding box. The distance could be derived from the dimension of the bounding box and the ratio of rgb peak. However, these AI approaches must be analyzed for effectiveness.

## Conclusion

This article introduces a novel approach of 4D sensor perception. This allows to process 4D motion through sensor information in relativistic image processing in order to relate spatial and temporal correlations with image and sensor information. To represent a 4D space, we introduced the Schlingal diagram. It describes space- and time-dependent information by 10 different DoF consisting of 4 translations and 6 rotations. Furthermore, we introduced the approach of a cross-dimensional metric to process information in a cross-dimensional way. This new approach supplements the use of sensor and image data for depth estimation and enables the prediction of motion.

We introduce the detection of features in superimposed images as a result. This technique emphasizes contours more strongly and favors the feature density. The FAST algorithm is particularly suitable for this approach. We also present the temporal-based search of corresponding features. The geometric relationships of features and the existence of temporal intersections can be detected by superimposing images of a scene. The geometry of the cross-dimensional metric serves as an auxiliary construct. Furthermore, we present the method of extracting sensor maps. This allows us to represent a relationship between sensor data and color gradients of a sensor map. The corresponding intensities of a respective sensor are expressed as a peak in the histogram. Our results show that object motions can be mapped as green components. This opens up the possibility of determining distances using measurable color components within a measured peak.

The promising results of our 4D model motivate us to continue our research on relativistic image processing by expanding further approaches in future work. We intend to validate our model for robotic and computer vision tasks by different sensor types. The sensor data shall be included under different environmental conditions to calculate 4D trajectories and image processing-based sensor maps. This includes the validation of sensor inaccuracies, dysfunctions, noise behavior, and integration errors on 4D calculations.

The connection between temporal-based correspondences, and cross-dimensional metric is an interesting part of depth estimation in relativistic image processing. Therefore, the algorithms will be extended in the near future to render point clouds from the temporal-based correspondences. In addition, the research on sensor maps shall be continued. This involves investigating further correlations and validating the transferability to other sensor data. The environmental scope of data also needs to be expanded for this purpose.

## Data Availability

All data generated during this study are included in this published article.
